# Angle‐Multiplexed 3D Photonic Superstructures with Multi‐Directional Switchable Structural Color for Information Transformation, Storage, and Encryption

**DOI:** 10.1002/advs.202400442

**Published:** 2024-05-17

**Authors:** Tao Wang, Yu Wang, Yinghao Fu, Zhaoxian Chen, Chang Jiang, Yue‐E Ji, Yanqing Lu

**Affiliations:** ^1^ National Laboratory of Solid State Microstructures Key Laboratory of Intelligent Optical Sensing and Manipulation College of Engineering and Applied Sciences and Collaborative Innovation Center of Advanced Microstructures Nanjing University Nanjing 210023 China

**Keywords:** 3D Photonic crystal, specular/diffuse reflection, structural color, silk protein, 1D folding

## Abstract

Creating photonic crystals that can integrate and switch between multiple structural color images will greatly advance their utility in dynamic information transformation, high‐capacity storage, and advanced encryption, but has proven to be highly challenging. Here, it is reported that by programmably integrating newly developed 1D quasi‐periodic folding structures into a 3D photonic crystal, the generated photonic superstructure exhibits distinctive optical effects that combine independently manipulatable specular and anisotropic diffuse reflections within a versatile protein‐based platform, thus creating different optical channels for structural color imaging. The polymorphic transition of the protein format allows for the facile modulation of both folding patterns and photonic lattices and, therefore, the superstructure's spectral response within each channel. The capacity to manipulate the structural assembly of the superstructure enables the programmable encoding of multiple independent patterns into a single system, which can be decoded by the simple adjustment of lighting directions. The multifunctional utility of the photonic platform is demonstrated in information processing, showcasing its ability to achieve multimode transformation of information codes, multi‐code high‐capacity storage, and high‐level numerical information encryption. The present strategy opens new pathways for achieving multichannel transformable imaging, thereby facilitating the development of emerging information conversion, storage, and encryption media using photonic crystals.

## Graphical abstract

Protein‐based 3D photonic superstructures capable of multi‐directional structural color imaging are developed. Embedding programmable designed light diffusion layers into a reconfigurable 3D photonic crystal enables multi‐image integration using specular and anisotropic diffuse reflections as different storage channels. The ability to program the platform's assembly configuration allows for multimode image switching, offering opportunities for developing information transformation, storage, and encryption devices.

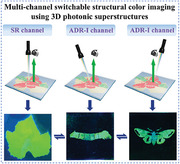

## 1 Introduction

With the advent of the information age, the convergence of factors such as digital transformation, widespread Internet access, the era of big data, and the advancement of the Internet of Things (IoT) has driven the rapid expansion of digital information. To manage this expansion and ensure the safe, secure, and efficient utilization of digital information, there is an urgent need for vast and manipulable storage space to store, manage, and transform information, as well as an increased demand for robust data security and privacy protection measures. To this end, diverse advanced information transformation, storage, and encryption strategies have been developed.^[^ ^1^ ^]^ Among the various strategies, optical imaging devices that can simultaneously accommodate multiple image information and dynamically transform them to deliver desired images on command have proven highly effective in high‐capacity storage and high‐security optical encryption applications. To date, extensive efforts have been made to investigate novel approaches to achieve integrated, dynamic, and switchable imaging capabilities in a single device to satisfy this criterion.^[^ ^2^ ^]^ Optical multiplexing is a newly developed information integration technology that makes use of the spatial distributions of light (e.g., angle, polarization, phase, and amplitude) to facilitate the combination of many information channels into a single device.^[^ ^2^, ^3^ ^]^ This technology provides an efficient, high‐capacity, and long‐lasting method for encoding, storing, transforming, and concealing multiple image information, and therefore has spawned the extensive development of a range of novel storage and encryption devices with distinct optical functionalities.

Until now, substantial progress has been achieved in creating optical multiplexing leveraging various optical phenomena, such as structural color, holography, and luminescence. Among others, structurally colored platforms, which depend on the delicate interaction of periodic nanostructures with incident light of a certain wavelength to obtain colorful characteristics,^[^ ^4^ ^]^ have drawn a lot of interest in this regard due to their ability to produce fading‐resistant, brilliant, tunable, and high‐resolution color displays. Existing structurally colored systems harness a range of photonic structures, including metasurfaces, diffractive gratings, and photonic crystals, to accomplish the multiplexing of information. Metasurfaces, consisting of arrays of subwavelength nanostructures, are renowned for their capacity to precisely manipulate amplitude/spectral and phase response of light for structural color and holographic functionality, respectively.^[^ ^5^ ^]^ Metasurfaces have been extensively explored for creating multiplexing holography that allows the generation of multi‐channel holographic images by incorporating various multiplexing methods such as wavelength,^[^ ^2^, ^6^ ^]^ polarization,^[^ ^7^ ^]^ and orbital angular momentum.^[^ ^8^ ^]^ Multiplexed structural color platforms have been proposed by encoding distinct images into different polarizations of a plasmonic or dielectric metasurface,^[^ ^9^ ^]^ enabling the manipulation of image information through precise control of these lighting parameters. While highly effective, this strategy often entails intricate micro‐nano structure designs, cumbersome manufacturing processes, and a reliance on specific equipment. Given their excellent structural and optical anisotropy, diffractive gratings offer another straightforward and efficient method for constructing angularly multiplexed structural color systems.^[^ ^10^ ^]^ However, the multispectral color display stemming from the inherent angle dependence of grating structures poses significant issues for application scenarios requiring consistent and reliable color representation.

Photonic crystals, particularly self‐assembled 2D and 3D colloidal photonic crystals, have emerged as highly promising platforms for generating dynamic, switchable structural colors owing to their scalability, simple processing, compatibility with a diverse range of materials and stimuli, and seamless integration with various top‐down manufacturing techniques.^[^ ^11^ ^]^ By enabling multimodal control over light‐nanostructure interactions through hierarchical manufacturing or integrating different photonic elements, photonic crystal systems can achieve multichannel image‐switching capabilities. They are capable of displaying distinct image information by appropriately modifying lighting parameters, such as incident angle,^[^ ^7b,d^ ^]^ polarization,^[^ ^12^ ^]^ direction,^[^ ^7^, ^13^ ^]^ and focusing plane.^[^ ^14^ ^]^ Additionally, combining photonic crystals with luminescent components to construct a multi‐optical component system provides a powerful solution for multichannel multiplexing.^[^ ^2^, ^15^ ^]^ While significant advancements have been made by these systems, constructing optically multiplexed photonic crystal systems with high‐capacity multi‐image integrations and the capability for crosstalk‐free switching between them still poses a formidable challenge. This challenge primarily stems from the difficulties associated with independently manipulating the interaction of light with photonic structures across multiple degrees of freedom.

Here, we describe the construction of angle‐multiplexed photonic superstructures based on programmable combinations of 1D quasi‐periodic folding structures and 3D photonic crystals that enable independent multi‐pattern storage, spatial‐angle‐dependent pattern display, as well as quick, stable, and precise multichannel pattern transformation. The embedding of 1D quasi‐periodic folding topography enables the unique combination of specular and anisotropic diffuse reflections within the photonic superstructure, each of which can be independently manipulated, providing distinct channels for structural color imaging. We demonstrate that, it is possible to programmablly integrate several mutually independent patterns—even those with great resolution—within a single system by engineering the structural assembly and topographical morphology of the photonic superstructure and to retrieve them separately devoid of crosstalk by only varying the angles of light illumination. We offer several demonstration devices to demonstrate this highly integrated imaging system's utility in information processing. We highlight its potential to multimodally change information and to store it in high‐capacity and highly secure encryption.

## 2 Results and Discussion

### 2.1 Angle‐Multiplexed Photonic Superstructure

**Figure 1:**
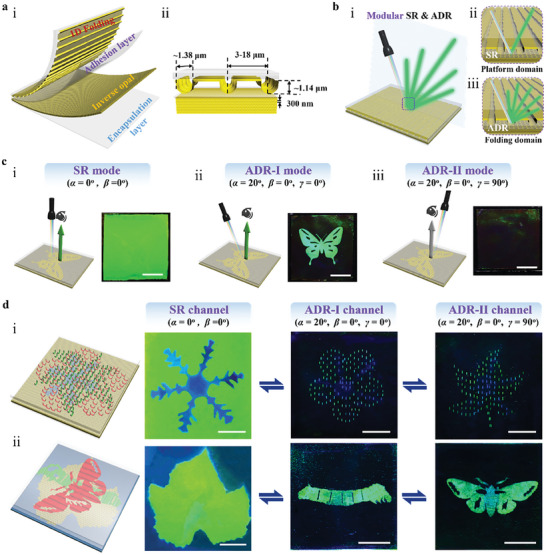
Structure and optical response of photonic superstructure and integrated transformable imaging device. a) Schematic illustration of the multiple structural layers of the angle‐multiplexed photonic superstructure (i) and the geometric dimension of each component (ii). b) Schematics showing the interaction between light and the photonic superstructure. The photonic superstructure merges independently manageable specular reflection (SR) and anisotropic diffuse reflection (ADR). c) Schematics (top) and Photographs (bottom) of the photonic superstructure with a templated anisotropic folding surface at various illumination‐viewing modes: specular mode (i), diffusive modes with incident light perpendicular (ii) and parallel (iii) to the folding orientation. Scale bars: 1 cm. d) (Left) Schematics of two photonic devices capable of multi‐directional image displays created by integrating patterned SIO with an orthogonally oriented folding surface (i) or with two anisotropic folding surfaces that overlap orthogonally (ii). (Right) Photographs of resulting devices under various lighting channels. Three different types of pattern information can be distinguished from the device by simply adjusting the direction of illumination. The definitions of *α*, *β*, and *γ* are shown in Figure S2, Supporting information. Scale bars: 1 cm.

According to **Figure** **1**a, the angle‐multiplexed photonic superstructure is a multilayered structure with the basic configuration of a bottom polydimethylsiloxane (PDMS) encapsulating layer, a silk inverse opal (SIO) layer, a thin PDMS adhesion layer, and a 1D quasi‐periodic folding surface created from a silk/PDMS bilayer. The inverse opal layer, which serves as a photonic crystal lattice layer, produces iridescent (angle‐dependent) structural colors that can only be distinguished in the area of the incident light's specular direction (Figure S1, Supporting Information). The 1D quasi‐periodic folding structure performs as an efficient anisotropic optical diffuser, enhancing the diffusion of light reflected from the underlying SIO in the plane perpendicular to the folding orientation. Silk protein extracted from native *Bombyx mori* silkworm fibers is utilized as the primary building blocks of the superstructure because of its unique ability to generate 1D quasi‐periodic folding structures with distinctive morphologies, as elaborated below. The utilization of silk protein also facilitates the generation of high‐quality photonic nanostructures and, thus, superior optical functions, through leveraging its mechanical robustness, nanoscale processability, and favorable optical properties.^[^ ^16^ ^]^ Furthermore, silk protein allows for the reconfiguration of topographical morphologies in both folding patterns and photonic lattices, by taking advantage of its controllable polymorphic transitions.^[^ ^7^, ^17^ ^]^ This capability enables the modulation of the spectral response of the final superstructures. As this photonic system constitutes a multilayer coupled structure, its assembly form can be tailored on demand, leading to the creation of a programmable integrated photonic superstructure.

Exploiting the distinctive topography of the folding surface (Figure 1a‐ii), characterized by anisotropy, quasi‐periodicity, high aspect ratio, and wide flat spacing between the two folds, this composite photonic structure combines independently controllable specular reflection and wide‐angle anisotropic diffuse reflection in a single device (Figure 1b‐i). The wide flat morphology between the two folds predominantly gives rise to specular reflection (Figure 1b‐ii), while the 1D aligned fold morphology contributes to diffuse reflection (Figure 1b‐iii). These capacities enable the controlled manipulation of the interaction between light and structure in 3D space as well as the production of various optical responses to white light under various spatial incidence situations. The folding surface on top has less of an impact on the optical signal reflected in specular mode due to the significant specular reflection of SIO at the stopband wavelength. In contrast, the anisotropic folding and SIO structures work together to produce the signal that is reflected in diffusive mode. Photographs of the photonic superstructure with a butterfly folding pattern taken in several illumination‐viewing modes (i.e., under normal viewing but various illumination conditions) are displayed in Figure 1c (see also Movie S1, Supporting Information). Under normal incidence illumination (specular illumination mode, *α* = 0°, *β* = 0°, Figure S2, Supporting Information), the photonic superstructure appears uniform and brilliant green colors in both folding and fold‐free areas. Under diffuse illumination, however, the device exhibits a striking butterfly pattern when the incident light is perpendicular to the folding orientation (*γ* = 0°), but it does not reflect any color when the light is parallel to the folding orientation (*γ* = 90°). The resulting device has optical stability and mechanical flexibility. It is easily bent and, whether bent inwards or outwards, consistently displays the butterfly pattern (Figure S3, Supporting Information).

In combination with the ability to reconfigure the architectures of folding structures and photonic lattices as well as to engineer their functional coupling, this angle‐dependent contribution from folding surfaces makes it possible to encode various patterns in the same area of the device and display them separately by simply changing the light illumination direction. In specular reflection mode, the device displays a multispectral SIO pattern; in diffusive mode, it displays a pattern formed from a templated folding structure with the incident light direction perpendicular to the folding orientation. In particular, the formation of several angular channels in diffusive mode is supported by the capacity to converge folding structures with prescribed orientations. This might be accomplished by either combining 1D folding strips with various orientations into a single bilayer or by building numerous 1D folding bilayer systems at various horizontal orientation angles. A proof‐of‐concept illustration for multichannel pattern displays based on these two methods is shown in Figure 1d. The first demonstrator device shows how triple‐channel image displays and transformation work by combining silk/PDMS bilayer with orthogonally oriented folding strips (Figure S4, Supporting information) and patterned SIO (Figure 1d–i). When the device is illuminated at normal incidence (*α* = 0°, *β* = 0°), the snowflake pattern is visible, while at an incidence angle of 20° (*β* = 0°), sakura and maple leaf patterns become visible at their respective encoding angles (*γ* = 0° and 90°). Another efficient method for triple‐channel picture displays involves orthogonally overlapping two templated anisotropic folding bilayers and incorporating them with patterned SIO. By merely changing the lighting direction, it is possible to switch back and forth between the mulberry leaf, silkworm, and moth patterns, as seen in Figure 1d‐ii. Unless otherwise specified, the incidence angle (*α*) and viewing angle (*β*) for all image observations are maintained at 20° and 0°, respectively.

### 2.2 Formation of 1D Quasi‐Periodic Folding Surface

**Figure 2:**
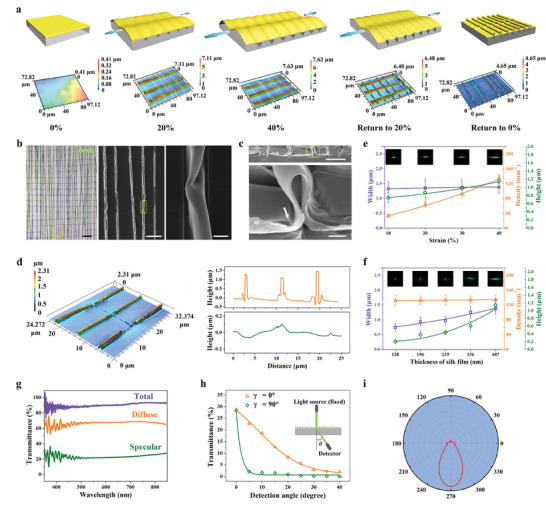
Fabrication of 1D quasi‐periodic folding pattern. a) (Top) Schematic of preparation of anisotropic folding pattern. Optical‐grade silk solution is spin‐coated on a soft PDMS substrate to form a silk/PDMS bilayer structure. Anisotropic folding is formed after stretching and subsequent releasing. (Bottom) Corresponding 3D laser‐scanning confocal microscope images showing the surface morphologies under various applied strains. Scale bar: 50 µm. b) Optical microscopy image (left) and surface SEM images (middle and right) of a typical folding surface (Strain: 40%). Scar bars (from left to right): 50 µm, 10 µm, 1 µm. c) Cross‐sectional SEM images showing the Ω‐shaped geometry of the folding structure. Scar bars: 5 µm (top), 500 nm (bottom). The arrow indicates a significant reduction in the thickness of the silk layer near the folding edge. d) (Left) 3D laser‐scanning confocal microscope image of the folding surface. (Right) Cross‐section profiles of the longitudinal (top) and transverse (bottom) folds. e,f) Experimental results of the folding's width, height, and density with various strains (e) and thicknesses of silk film (F). Insets show the corresponding transmitted diffraction patterns generated by using a green laser (λ = 543.5 nm) illumination. g) Total, specular, and diffuse transmittance spectra of the folding surface. h) Dependence of transmittance on the detection angle under diffusive transmission mode (wavelength: 550 nm). The inset shows the diagram of a diffusive transmission measurement system. i) The numerically calculated far‐field pattern of the anisotropic folding structure.

The method for creating a 1D quasi‐periodic folding pattern is based on a straightforward bilayer system made of a soft PDMS substrate and a very thin layer of stiff silk spun on top of it. After uniaxial stretching and subsequent release of the bilayer structure, the mechanical imbalance between silk and PDMS causes the creation of a folding surface. **Figure** **2**a depicts how the surface topography of the bilayer system changed as the folding pattern took shape. Due to the brittleness of the surface oxidized layer, when mechanical force is applied, quasi‐periodically distributed cracks in the PDMS layer that are perpendicular to the stretching direction develop (see also Figures S5 and S6, Supporting Information).^[^ ^18^ ^]^ Meanwhile, the thin silk layer experiences elastic‐plastic deformation, with the layer that is located atop the crack openings absorbing the majority of the deformation by becoming thinner in thickness. As a result of the constant rise in applied strain, the number, width, and depth of cracks increase (Figure 2a; Figures S6‐S8, Supporting Information). In contrast, the thickness of the silk layer decreases. In the meantime, the compressive force brought on by Poisson's effect causes regular wrinkles to appear parallel to the strain (Figure 2a).^[^ ^19^ ^]^ The transverse wrinkles gradually become less prominent as the mechanical strain is gradually relieved, the cracks tend to close, and the plastically deformed silk layer on top of them folds via a mechanically guided compressive buckling process (Figure 2a; Figure S9, Supporting Information).^[^ ^20^ ^]^ After the strain is released, anisotropic folding structures are eventually formed, and the wrinkling topography goes back to its original flat state. In contrast to previously reported mechanically sensitive bilayer materials,^[^ ^21^ ^]^ which often produce anisotropic cracks during/after mechanical stretching, the development of the 1D quasi‐periodic folding patterns exhibited here is unique. This unique behavior is due to the thin silk film's remarkable mechanical ductility and the “moderate” adhesion between silk and PDMS. While the latter enables the silk layer to be detached from the PDMS crack holes, the former ensures the elastic‐plastic deformation of the silk layer during stretching. It is also interesting that neither during nor after applying mechanical tension do cracks appear on the silk layer.

The surface microstructure of a typical folding system is shown in Figure 2b, which amply demonstrates the 1D quasi‐periodic arrangement of folds separated by flat surfaces. The fold density, which is calculated as the number of folds divided by the fold‐to‐fold distance (Figure S10, Supporting Information), is around 134 ± 12 mm^−1^. The width of the folds is approximately 1.38 ± 0.20 µm. Cross‐sectional images reveal that the folding structure consists of a silk‐thin layer alone with an Ω‐shaped geometry (Figure 2c). The height of the folds can be determined using a 3D laser‐scanning confocal microscope image, which shows a measurement of ≈1.14 ± 0.16 µm (Figure 2d). Notably, the mismatch in stiffness between silk and PDMS causes transverse periodic folds to form along the wrinkle valleys as a result of mechanical actuation,^[^ ^21a^ ^]^ as shown in Figure 2a,b. Due to their low aspect ratio (Figure 2d; Figure S11, Supporting Information), we discovered that these lateral folding patterns scarcely scatter light (details below). As a result, we represent the surface topographical morphology as 1D quasi‐periodic folding patterns and ignore these transverse folds. By varying the applied strain and the silk layer thickness, the geometrical parameters of the folding structure can be changed. As shown in Figure 2e, when the applied strain increases, the height and density of the folding rise but the width generally remains the same (see also Figures S12‐S14, Supporting Information). The height and width of the folding gradually grow as the silk layer's thickness rises, while the density stays essentially constant (Figure 2f; Figures S15‐S18, Supporting Information). In addition, for a given silk layer's thickness, the spacing between two folds shows a normal distribution, and it remains constant regardless of the thickness of the silk layer (Figure S19, Supporting Information).

By redistributing the specular and diffuse components of the total transmittance, the surface folding patterns constitute an efficient way to control light transmission. The bilayer exhibits a low diffuse transmittance of 7.76% (at 550 nm) and a high specular transmittance of 89.10%, which is close to the total transmittance of 96.87%, in the absence of folding (Figure S20, Supporting Information). However, when the silk layer is folded under a 40% strain, the bilayer exhibits a high diffuse transmittance of 67.53% and a moderate specular transmittance of 29.17%, illustrating an improved light scattering of the folding surface (Figure 2g). Additionally, anisotropic light diffusion is produced by this 1D quasi‐periodic arrangement of the folded surface topography. A narrow band diffraction pattern with the long axis parallel to that of the applied strain is shown on the projected screen after illuminating a typical folding sample with a green laser (Inset in Figure 2h). The measurement of the diffuse transmittance spectra supports the anisotropic effect (Figure S21, Supporting Information). As shown in Figure 2h, the diffuse transmittance decreases gradually as the detection angle (*β*) increases from 0° to 40° with the horizontal orientation angle (*γ*) at 0°, while it drops dramatically with the detection angle increasing from 0° to 5° for *γ* = 90°. The finite element method depicted in Figure 2i theoretically supports the diffuse transmission effect. In line with the findings of the experiment, the far‐field numerical calculation of the folding surface reveals that the diffuse transmission angle spans from 230° to 310° and the intensity diminishes with increasing the transmission angle. Additionally, this anisotropic effect can be precisely modulated by varying the applied strain or silk layer thickness stated above, which enables the capacity to regulate light diffusion/diffraction on demand. As shown in insets in Figures 2e,f, enhanced light‐scattering was observed with the increase in applied strain or silk layer thickness.

### 2.3 The Optical Performance of the Photonic Superstructure

An iridescent structural color that can only be seen at a specific angle of specular reflection is typically produced by photonic structures with periodic layouts,^[^ ^4^, ^7^ ^]^ such as 3D photonic crystals. Through their functional interaction, an anisotropic folding surface and a periodic photonic structure have the potential to modify the final structures' overall structural color look. Particularly, the diffusely reflected structural color in the direction orthogonal to the folding orientation can be greatly enhanced by the anisotropic folding topography. To do this, we created silk‐based 3D photonic structures (SIO) by using polystyrene colloidal crystal multilayers as templates, as previously mentioned (Figure S22, Supporting Information).^[^ ^7^, ^17^ ^]^ The folding surface was eventually attached to the nanostructured side of the SIO using PDMS as the adhesive layer, completing the fabrication of the photonic superstructure.

**Figure 3:**
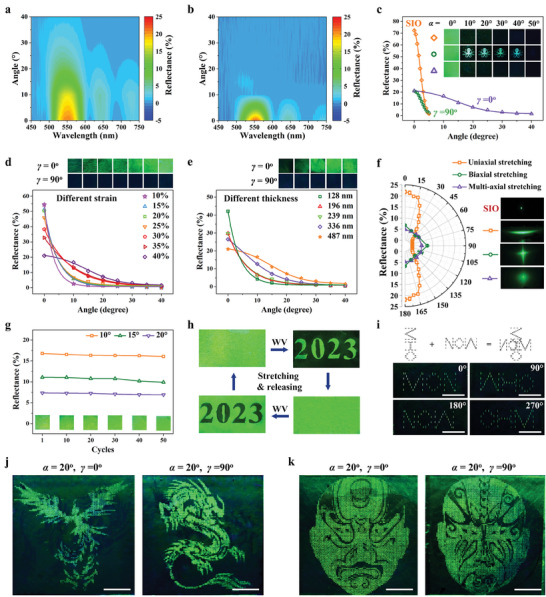
Optical properties of the photonic superstructure. a,b) Contour maps of the reflectance spectra of the photonic superstructure measured under diffusive reflection mode at different *β* values (a: *α* = 0°, *γ* = 0°; b: *α* = 0°, *γ* = 90°). c) Dependence of reflectance intensity of the stop‐band peak on the detection angle. Inset shows the photographs of reflected structural coloration on the SIO and the photonic superstructure with an octopus folding pattern at various *α* values. d,e) (Top) Photographs of the photonic superstructures with various strains (d) and thicknesses of silk film (e) of the folding structure captured at *α* = 20°. (Bottom) Corresponding reflectance intensity as a function of *β* (*α* = 0°, *γ* = 0°). f) (Left) Reflectance of the photonic superstructures with different folding orientation modes as a function of *γ* (*α* = 0°, *β* = 10°). (Right) Corresponding reflected light diffraction patterns illuminated by a green laser. g) Reflectance variation of the photonic superstructure upon 50 stretching and releasing cycles of the folding components. Insets are the corresponding photographs. h) Responsive behavior of the photonic superstructure to water vapor (WV) exposure, showing a “reversible erasing and rewriting” feature. i) (Top) Drawing designed to create a combination of different letters. (Bottom) Photographs showing the display of “MON, WHO, OHM, NOW” patterns at various *γ* values. j,k) Photographs showing the switching between the dragon pattern and the phoenix pattern (j) and between Beijing Opera facial makeup patterns (k). Scale bars: 1 cm.

We observed angle‐resolved reflectance spectra in diffusive mode at various detection angles (*β*) to examine the light reflection caused by the folding structure. As seen in **Figure** **3**a‐c, when the horizontal orientation angle (*γ*) is 0°, the reflected stop‐band peak, which is centered at *λ* = 550 nm, gradually loses intensity while keeping wavelengths relatively constant as *β* increases from 0° to 40° (*α* = 0°) (see also Figure S23a,b, Supporting Information). This suggests a wide detection range (−40°–40°), which is consistent with the folding system's transmittance measurements. However, the reflectance peak dramatically lowers its intensity as *β* increases from 0° to 5° for *γ* = 90° (see also Figure S23c,d, Supporting Information), similar to the angular response behavior of bare SIO (see also Figure S24, Supporting Information). These spectrum results support the folding structure's anisotropic light‐diffusing property (Figure 2i), which results in a wider range of detection/viewing angles for the color canvas beneath. We developed a photonic superstructure with an octopus folding pattern to illustrate this improved visibility. As the incidence angle is extended to 40° at *γ* = 0° (Inset in Figure 3c), the vivid green octopus pattern is still discernible as opposed to the photonic superstructure at *γ* = 90° or SIO, which only displays specular structural color. Due to the special folding topography (Figure 2b) that enables the coexistence of modular specular and diffuse reflections (Figure 1b), the addition of the folding surface, surprisingly, does not result in a glaring change in the color appearance of the specular mode (Inset in Figure 3c, *α* = 0°).

Due to the folding geometry's impact on light diffusion as stated above, it is possible to adjust the reflection characteristics (or structural colors) of composite photonic structures at various angles. As seen in Figure 3d,e, decreasing specular reflectance and increasing diffuse reflectance are caused by increasing the applied strain or silk layer thickness (see also Figures S25 and S26, Supporting Information). Correspondingly, when light is illuminated from the direction vertical to the folding structure (*γ* = 0°), the color brightness observed in diffusive mode increases with an increase in strain or thickness. On the other hand, when light is illuminated parallel to the direction of folding (*γ* = 90°), all the samples appear black. We also looked at the superstructure's diffuse reflection at various horizontal orientation angles and contrasted it with the situation in which the light‐diffusing layer was made up of an orthogonally or randomly oriented folding system (Figure S27, Supporting Information). The superstructure with anisotropic folding patterns exhibits progressively decreasing reflectance with an increase in *γ* from 0° to 90°, which is consistent with the diffraction pattern (Figure 3f; Figure S28, Supporting Information). The superstructure with orthogonal folding patterns exhibits the greatest reflectance at 0° and 90° (Figure 3f; Figure S29, Supporting Information), while the one with randomly oriented folding patterns exhibits isotropic reflection (Figure 3f; Figure S30, Supporting Information). We determined the viewing angle of the anisotropic photonic superstructure at *γ* = 0° to be ≈47.86° (Figure S31, Supporting Information) based on the reflected light diffraction, which is comparable with the spectral analysis result shown in Figure 3c.

Thanks to the mechanical flexibility and robustness of the silk/PDMS bilayer system, which enables the formation of a stable folding topography during numerous stretching/releasing cycles (e.g., 50 cycles, Figure S32, Supporting Information), the composite photonic structure exhibits excellent stability in light scattering with minimal variations in reflectance (or structural color) during repeated stretching/releasing of the folding structure (Figure 3g; Figure S33, Supporting Information). Moreover, the photonic superstructure maintains consistent reflection properties even after being stored at ambient conditions (20‐30 °C and 30–60% RH) for 12 months, demonstrating its excellent long‐lasting stability (Figure S34, Supporting Information). The folding structure and the photonic lattice (of SIO) can both be reconfigured by external stimuli, such as water vapor, which can cause the molecular chain movement of silk protein, even though the composite system is stable in ambient settings. This tactic takes use of the amorphous silk's polymorphic transition and has been demonstrated to allow for the controllable reconfiguration of the photonic nanostructure of bare SIO.^[^ ^17a^ ^]^ By being exposed to water vapor, the folding topography discussed here can be removed, leaving behind a smooth surface (Figures S35 and S36, Supporting Information). When the folding patterns are removed, the composite system's diffuse reflection is decreased, while the specular reflection is increased (Figure S37a,b, Supporting Information), which causes the bright green color to turn black when viewed in a diffusive mode. The unfolded bilayer can be treated once again by mechanical stretching to create folding patterns that exhibit a similar reflection to the first one (Figure S37c, Supporting Information). This transition process is reversible for more than 10 cycles. Rewritable pattern designs can be created by carefully applying water vapor using various shadow masks. Applying strain, for instance, can reverse a “2023” pattern by erasing the initial writing on the surface and returning it to its folded state (Figure 3h).

By controlling the orientation of folding structures, it is possible to encode different optical messages at various incident angles because the range of reflection can only converge in the plane orthogonal to the folding orientation in diffusive mode. By arranging orthogonal silk narrow strips on a PDMS surface and applying biaxial stretching, we discovered that anisotropically aligned folding structures with directions perpendicular to the long axis can develop in each unit (Figure S4a, Supporting Information). Following integration with SIO, this enables the encoding of independent and different patterns at orthogonal angles (Figure 3i; Figure S4c,d, Supporting Information). Moreover, two folding structures with the proper folding geometry can be orthogonally overlapped to produce the superposition of different reflection information, which is then integrated with SIO (Figure S38, Supporting Information). This offers a simple method for the device to display intricate, high‐resolution patterns in two mutually perpendicular illumination directions. We used a laser engraving technique to generate dot matrix designs of a phoenix and a dragon on two folding bilayers, which were then merged with SIO in an orthogonal configuration to show the level of pattern complexity that is possible. In diffusive mode, the device exhibits a dazzling phoenix pattern when illuminated perpendicular to its folding orientation, and the dragon pattern when rotated 90 degrees horizontally, as illustrated in Figure 3j and Movie S2, Supporting Information. Additionally, it is shown that transitioning between two different face makeup styles imitates the well‐known Beijing Opera Face Changing (Figure 3k). It is worth noting that the reflectivity of the photonic superstructure decreases as the number of stacked layers increases (Figure S39, Supporting Information), which slightly reduces the clarity of each piece of information. We believe that by optimizing the assembly structure and integration method of multiplexed photonic crystals, it is possible to improve their reflection efficiency. Following the reflectivity results of the photonic superstructure at various *γ*, the theoretical upper limit of multiplexed images is five (one image in the SR channel and four images in ADR channels). Although the photonic superstructure system developed here is not directly comparable to the systems based on multiplexed holographic technologies in terms of information capacity, its capacity is significantly improved compared to existing photonic crystal‐based multiplexing systems.

### 2.4 Multi‐Directional and Multimodule Imaging

**Figure 4:**
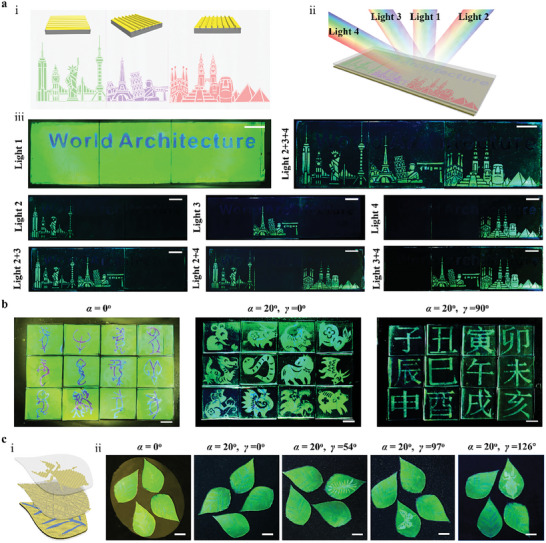
Multi‐directional and multimodule imaging functionalities. a) (i, ii) Schematic illustration of the design of a world's famous architectural pattern featuring three different folding orientations (i) and four types of illumination schemes applied to the integrated device with each light beam covering the entire device area (ii). (iii) Photographs of the resulting device under various light illumination modes. Scale bars: 1 cm. b) Photographs of a “modularized” imaging device, showing the switching between the oracle bone inscriptions of twelve Chinese zodiacs, the animal patterns, and the corresponding twelve terrestrial branches. Scale bars: 1 cm. c) (i) Schematic to show the design of a bionic camouflage photonic device. The device is a three‐layer structure consisting of a leaf‐shaped, patterned SIO, a quasi‐isotropic folding structure, and an anisotropic, templated folding structure. (ii) Photographs showing the separate visualizations of the caterpillar, butterfly, and leaf insect pattern resting on the leaves. Scale bars: 1 cm.

The ability to program both the folding topographies and photonic nanostructures facilitates the storage of diverse distinct patterns in a specular reflection channel and multiple diffuse reflection channels, as demonstrated in Figure 1e. This capacity, combined with the possibility to engineer the form in which photonic superstructure can be assembled, allows the angle‐multiplexed photonic systems to accomplish various forms of multi‐directional imaging. We first created a famous architectural design that allows the on‐demand display of constituent elements in various illumination modes as a demonstration. This required segmenting the pattern into three separate sets of components, each of which was then processed in a unique way to permit various folding structure orientations (**Figure** **4**a‐i). These parts were put together in a prescribed sequence onto the “World Architecture”‐patterned SIO. Building on this concept, the integrated device was dynamically applied with one vertical illumination and three oblique illuminations, or a combination of them, to form a variety of images (Figure 4a‐ii). When vertical irradiation is applied (*α* = 0°, Light 1), only the “World Architecture” pattern is visible (Figure 4a‐iii). However, the integrated device enables the observation of individual elements or paired combinations of famous architectural images when the partial oblique incidence of various orientations is selectively applied. Furthermore, the full architectural patterns are revealed, providing a comprehensive visual display, when all oblique incidences are applied simultaneously (Lights 2+3+4). A striking feature of this angle‐multiplexed photonic device is that it enables quick, stable, and on‐demand switching of several pictures as well as their crosstalk‐free exhibition. It should be emphasized that this remarkable quality is achieved under the condition that each beam of light illuminates the gadget entirely and uniformly, without the need to deliberately illuminate each component separately. Another example is creating a “modularized” imaging device, where each module comprises two orthogonally superimposed templated folding systems and a patterned SIO (see Experimental Section for details). As seen in Figure 4b, by merely changing the light's illumination direction, the oracle bone inscriptions of the twelve Chinese zodiacs, the animal patterns, and the corresponding twelve terrestrial branches can be switched back and forth (see also Movie S3, Supporting Information).

Moreover, by properly designing assembly structures, the integrated photonic devices are also appropriate for bionic camouflage because they can blend in with or stand out from the colors of the surroundings by hiding or disclosing folding pattern information by altering the light illumination state. As proof, we created a leaf‐shaped camouflage system to mimic the behaviors of some organisms in nature to conceal themselves. As shown in Figure 4c‐i, the system was assembled by combining the templated 1D folding structure with a folding structure that was randomly orientated and an SIO substrate with leaf vein patterns. By supplying quasi‐isotropic light diffusion, the embedding of a randomly oriented folding layer makes it possible to see the patterned SIO in both specular and diffusive modes. The integrated camouflage systems are deliberately positioned erratically with different angles to ensure individual identification of each camouflaged object. When observed in specular mode, the insects resting on the leaves are entirely hidden from the viewer, but when viewed in diffusive mode, they are each individually exposed with variations in the horizontal detecting angle (Figure 4c‐ii). Notably, this system operates in a passive mode that just depends on ambient lighting and doesn't require any additional stimuli, showing the possibility for highly effective adaptive visible camouflage.

### 2.5 Demonstrations for Information Transformation, Storage, and Encryption

**Figure 5:**
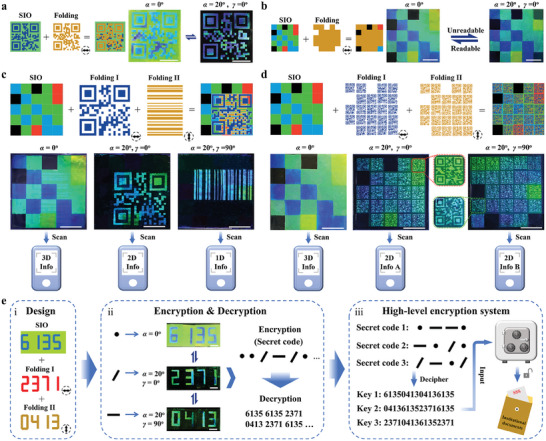
Use of the angle‐multiplexed photonic superstructures for multimodal information transformation, high‐capacity storage, and high‐level encryption. a) (Left) Schematic diagrams to show the design of the photonic devices for information transformation. The arrows in the lower right corner indicate the folding orientation. (Right) Photographs of the devices captured under different illumination modes. The device shows the transition between two different QR codes by changing the illumination angle *α* from 0° to 20° (*β* = 0^o^, *γ* = 0^o^). Scale bars: 1 cm. b) (Left) Schematic of photonic device design for transformation between readable and unreadable information. (Right) Photographs showing the transition from a readable 3D code to an erroneous 3D code. Scale bars: 1 cm. c,d) (Top) Schematics showing the organization of photonic devices for multichannel and multimode information storage. (Bottom) Photographs of the devices captured at different illumination modes, showing the information code stored in each channel that can be scanned by corresponding software. The enlarged images reveal the formation of QR codes with high quality. Scale bars: 1 cm. e) (i) Schematic diagram of the design of an encrypted photonic device with three different sets of numerical data. (ii) Encryption and decryption strategy based on the multilevel encrypted photonic device. (iii) High‐level encryption system for high‐security document protection. Scale bars: 5 mm.

The integrated photonic devices have significant potential for various information processing applications due to their ability to flexibly integrate multiple independent pieces of information into a single system and then decode them under varying lighting orientations. Examples that illustrate their capacity for multimodal information transformation, high‐capacity information storage, and high‐level encryption are shown in **Figure** **5**.

1D barcodes, 2D codes, and 3D color codes, employing parallel lines and spaces, arrays and matrices, and colored patterns to produce readouts of encoded information, have been widely used in many fields intimately tied to people's daily lives.^[^ ^17^, ^22^ ^]^ While these information codes can be useful for information storage and readout, the encoded information cannot be changed or manipulated after it has been created. The integrated photonic devices that resulted can encode different codes into different irradiation angles and directions, enabling the rapid, stable, and crosstalk‐free transformation between different codes. Figure 5a illustrates how changing the incidence angle (*α*) from 0° to 20° can cause a 2D code printed in the SIO to change into a 2D code written in the surface folding. Moreover, by changing the horizontal orientation angle (*γ*) from 0° to 90°, information translation between two 2D codes or between 1D and 2D codes can be accomplished (Figure S40 and Movie S4, Supporting Information). All these written codes can be successfully scanned without crosstalk. Based on this information transformation trait, the encoded information can be efficiently concealed inside the printed code in diffuse mode by lowering the local patterns' capacity to diffuse light. This feature allows the disclosure of encoded data only under specific conditions (specular reflection) and supports a reversible transformation between readable (or correct) and unreadable (or incorrect) information. To illustrate this concept, we created a scannable 3D code by exposing different portions of SIO to water vapor for varying lengths of time and then covered it with a templated folding surface that had been locally flattened by water vapor exposure (Figure 5b). The 3D code can solely be read out by a smartphone app upon 0° illumination. The concealing of color information beneath the fold‐free portions, however, causes the code to become unreadable when the illumination angle is sloped, such as at 20°.

Traditional information codes often only support a single type of information, and they have limited storage space. However, the study's description of multichannel code storage and reversible code‐switching offers a viable strategy for greatly increasing information storage capacity. We built a three‐channel information storage device as a proof‐of‐concept by orthogonally superimposing a folding surface with a 1D barcode pattern and another folding surface with a 2D code pattern, then combining them with a 3D code produced from SIO. As shown in Figure 5c, by correctly adjusting the horizontal orientation angle and the angle of light illumination, the 3D code, 2D code, and 1D code can each be independently obtained. After extraction, the associated software can be used to scan out the data contained within each code. As a result, the system successfully supports the simultaneous storage of 1D, 2D, and 3D information. We created two sets of 2D code arrays on two folding systems to further highlight the possibility of greater data storage capacity within a single protein‐based photonic device. We then integrated them with the 3D code generated by the SIO. As expected, with the right illumination and scanning, each piece of encoded information could be quickly and simply decoded (Figure 5d). The end device stores 44 different forms of 2D information together with one type of 3D information, demonstrating its ability to handle a diverse range of data. It is crucial to remember that the information code devices built on the described photonic superstructure show promising potential for achieving increased information storage density and more flexible information combinations by further improving written pattern resolution and extending encoding angles.

In addition, the use of integrated photonic devices for multichannel numerical information encryption becomes feasible by the need for particular lighting modes, orientations, and sequences for information retrieval. By constructing complex decryption procedures, the extracted numerical information can subsequently be used to design secret codes for the creation of a high‐security encryption system. As an illustration, we developed a photonic device that included three different sets of numerical data, namely “6135”, “2371”, and “0413” (Figure 5e‐i). A secret code for numerical data encryption can then be made using each of these sets of information that is only perceptible under a certain illumination direction (Figure 5e‐ii, and Movie S5, Supporting Information). Specifically, we designate “6135” as “•”, “2371” as “∕”, and “0413” as “–”. By arranging the symbols “•”, “∕”, and “–” in a flexible and arbitrary manner, we can effectively encrypt distinct information using this approach. For instance, a secret code such as “• • ∕ – ∕ •” can be employed to encrypt numerical data “613 561 352 371 041 323 716 135” (Figure 5e‐ii). We further created a high‐level encryption system for protecting secret documents using this encryption approach (Figure 5e‐iii). In this approach, the document was kept in a safe that had three locks, each of which was protected by a 16‐digit password. Each lock's password is encoded in a special secret code that can only be successfully decoded by a designated party who is in possession of the necessary decryption tools. This is accomplished by carefully manipulating the required order of lighting conditions, which finally results in the discovery of the special 16‐digit password assigned to each lock and the subsequent retrieval of the secured document. Due to its complex decoding procedure and the vast number of possible password combinations, this high‐level encryption system greatly increases the difficulty of decryption and improves information security, effectively reducing the risk of unauthorized access and improving overall data protection.

## 3 Conclusion

In summary, the combination of reconstructable 1D folding systems and reconfigurable 3D photonic crystals provides a dependable strategy for multi‐directionally transformable structural color imaging by converging independently controllable specular reflection and anisotropic diffuse scattering into a single protein‐formatted photonic system. The integrated photonic superstructures exhibit improved optical multiplexing capabilities by offering multiple degrees of freedom to manipulate light in 3D space, thereby enabling the generation of co‐located multi‐pattern storage, direction‐controlled crosstalk‐free pattern display, as well as efficient and reliable pattern switching. Beyond the demonstrators given here, the designable assembly form of the photonic superstructure along with the structural polymorphism and versatility of the protein format enables the creation of various sorts of intelligent, multipurpose, and adaptive information devices. Multi‐directional high‐resolution full‐color imaging would be attainable by refining the patterning approach of the 3D photonic crystal layer through strategies such as combining colloidal crystal self‐assembly with inkjet printing techniques.^[^ ^17^, ^23^ ^]^ By optimizing the integration modes to increase the number of angle channels or by including additional light‐responsive channels (such as fluorescent, phosphorescent, or holographic signals) into the current multiplexing platform, the capacity to manage multiple optical signals simultaneously could be further enhanced. These protein‐based multiplexing systems would hold promising potential for real‐world utilization across various domains, driven by the persistent expansion in silk material production scale and the ongoing improvement of relationships between structures, properties, and functionalities within silk optical systems. This study's multiplexing strategy opens new directions for applications such as high‐security encryption, high‐density optical storage, multimode information transformation, and bionic adaptive camouflage that uses photonic crystals. There are notable opportunities for extending this strategy to other iridescent systems, such as chiral liquid crystals, multilayer thin films, and plasmonic metasurfaces paving the way for exciting prospects in the future advancement of intelligent and integrated optical imaging devices.

## 4 Experimental Section

### Preparation of Silk Fibroin Solution

The regenerated silk fibroin solution was prepared according to established protocols.^[^ ^24^ ^]^ Briefly, raw cocoons were boiled in a 0.02 M Na_2_CO_3_ water solution for 30 min, and thoroughly rinsed with distilled water to remove the sericin layer. After drying for 2 days, the remaining silk fibroin fibers were dissolved by 9.3 M LiBr at 60 °C for 4 h. The dissolved silk fibroin was subsequently dialyzed against distilled water for 3 days, yielding a 6–7 wt.% silk fibroin solution in water. To obtain a concentrated suspension (14‐16 wt.%), the initial silk solution was gently blown with a fan to allow slow evaporation of water from the solution.

### Preparation of Silk/PDMS Bilayer Folding System

Elastic Poly (dimethyl siloxane) (PDMS) (Sylgard 184, Dow Corning) film with a thickness of ≈1 mm was prepared by mixing base/curing agent mixture in a 10:1 weight ratio and curing at 60 °C for 24 h. The cured PDMS film was cut into squares and then etched using a plasma cleaner (Zepto‐BLS, Diener electronic) at a pressure of 300 mTorr with 30 W for 60 s. Plasma etching was used to improve the adhesion stability of silk film on the PDMS substrate by inducing the formation of a very thin oxidized layer on the PDMS surface. To prepare the Silk/PDMS bilayer, a certain concentration (12 wt.%, if not otherwise indicated) of silk fibroin solution was spin‐coated on the etched PDMS substrate using a spin‐coater (KW‐4A, CHEMAT) at 40%−50% humidity with two steps: 500 rpm for 12 s and 3000 rpm for 60 s. To acquire a uniaxially oriented folding surface, the bilayer sample was unidirectionally stretched to 40% strain (if not otherwise mentioned) for 1 min using a custom‐built stretching tool and then released to its initial state (RH: 40%−50%). The resulting samples were stored in a dry box (RH: 35%) for the subsequent experiments. Bidirectionally or multi‐directionally stretched Silk/PDMS bilayers were obtained by following the stretching mode shown in Figure S24 (Supporting Information).

### Preparation of Silk Inverse Opal (SIO) Film

The SIO films were prepared by using polystyrene colloidal crystal multilayers as templates. The detailed fabrication process was described in previous works.^[^ ^17^, ^25^ ^]^ Briefly, 30 µL suspension (4 wt.%) of monodisperse polystyrene spheres (300 nm in diameter, modified by carboxylic acid group on the surface, Interfacial Dynamics Co., Ltd.) was introduced onto a water surface to form a floating monolayer. A large‐scale hexagonally close‐packed polystyrene monolayer array was formed at the water/air interface after removing the spheres sunk into the subphase of water and adding a few drops of sodium dodecyl sulfate. This self‐assembled monolayer was transferred from the water surface to the surface of a hydrophobic substrate (tridecafluoro‐1,1,2,2‐tetrahydrooctyl trichlorosilane (FOTS) treated silicon wafer). Colloidal crystal multilayers with desired layer numbers were obtained by repeating the transferring procedure. The silk fibroin was poured into the colloidal crystal templates to fill all the air voids, followed by drying for 24 h (25 °C, 30%−40% relative humidity) to form a free‐standing silk/polystyrene composite film. SIO film with a thickness of 50 µm was finally obtained by immersing the dried composite film in toluene for 24 h to remove the polystyrene spheres.

### Patterning of Folding Surfaces and SIOs

1) Templated Folding Surfaces
  (1) *Plasma Etching*: Initially, a fold‐free Silk/PDMS bilayer covered by a specially designed PDMS shadow mask was exposed to oxygen plasma (300 mTorr, 30 W) for 0.5 h. After removing the mask, the post‐treated bilayer was stretched to 40% strain and then released to induce the formation of folding topography. During oxygen plasma etching, the thin silk layer in the exposed region was selectively removed, while the covered area remained intact, thus resulting in the formation of a templated folding surface after stretching and releasing.
*Patterns Created using Plasma Etching*: butterfly in Figure 1c; silkworm and moth in Figure 1d‐ii; Octopus in Figure 3c; Chinese Zodiac in Figure 4b; baron caterpillar, leave insect, and butterfly in Figure 4c; QR codes in Figure 5a,c; templated folding in 5b; numbers in 5e; 2D code in Figure S40, (Supporting Information).
  (2) *Laser Engraving*: A CO_2_ laser cutter (SIR‐2075, Trotec) was used to create high‐resolution and complex patterns on the folding surface. Under engraving mode with controlled speed and power, the high‐energy laser induces the removal of the thin silk layer on the smooth Silk/PDMS bilayer in the engraved area. After the stretching/releasing process, folding topography was only formed in the unengraved area.
*Patterns Created using Laser Engraving*: maple leaf and sakura in Figure 1d‐i; letters in Figure 3i; dragon and phoenix in Figure 3j; facial makeup in Figure 3k; Landmark buildings in Figure 4a; 1D code in Figure 5c; QR codes in Figure 5d; butterflies in Figure S4, Supporting Information; 1D code in Figure S40, Supporting Information.
  (3) *Water Vapor Treatment*: Water vapor exposure was utilized to erase the folding patterns. PDMS shadow masks with specific designs were first covered conformally on the folding surface. The masked sample was then positioned close to a high humidity source (e.g., a heated water surface (about 40 °C)) to directly expose the folding surface to water vapor for a short period (≈5‐10 s). This erasure process is reversible. The erased regions can be restored to their initial folding state by a stretching/releasing process.
2) Patterned SIO Films

Water vapor or UV light treatment was used to design various patterns on SIO films. The process of water vapor exposure is the same as that for folding surface patterning with the exception that the treatment time is fixed at 3 s. UV germicidal lamps (G36T5VH, Serve Tool Inc.) with a wavelength of 254 nm and a power of 40 W were used for UV irradiation. Shadow masks with designed shapes were covered on the nanostructured surface of SIO before UV treatment to leave the desired pattern on it after mask removal. The distance between the sample and the UV lamp was fixed at 1 cm.

### Fabrication of Angle‐Multiplexed Photonic Superstructure

The fabrication process for the angle‐multiplexed photonic superstructures involved two main steps. Initially, the nanostructured surface of a SIO film was affixed to the folding surface of a Silk/PDMS bilayer, employing a slender PDMS adhesion layer (≈50 µm thick). Subsequently, this combined structure was enclosed by affixing an additional PDMS layer (≈200 µm thick) onto the non‐nanostructured facet of the SIO, with the edges tightly adhered to the PDMS layer of the folding bilayer.

### Structural and Properties Characterization

Optical microscopy images were captured using an optical microscope (DM 2700 M, Leica). Surface and cross‐sectional morphologies of folding surface and SIO were observed using a field emission scanning electron microscope (S‐8100, Hitachi, Japan) at a voltage of 5 kV and a current of 10 µA. 3D topographies of folding patterns were analyzed by a 3D laser‐scanning confocal microscopy (VKX1000, KEYENCE, USA). The silk film thickness of the silk/PDMS bilayer was measured by a surface profiler (Dektak‐XT, Bruker) with a maximum measurement range of 6.5 µm and a duration of 50 µm s^−1^. Photographs and Movies were taken with a digital single‐lens reflex (DSLR) camera (EOS 850D, Canon). Total and specular transmittance spectra were acquired by a Fiber‐optic spectrometer (PG2000‐Pro back‐thinned spectrometer, Ideaoptics, China). An integrating sphere (IS‐50‐10‐GT, Ideaoptics, China) was incorporated into the spectrometer to obtain total transmittance spectra. All angle‐dependent spectra were collected using an angle‐resolved spectrometer (R1, Angle‐resolved spectrum system, Ideaoptics, China). The angle‐resolved reflectance and transmittance spectra were measured by fixing the incident light (normal to the sample surface) and adjusting the detection angle with respect to the surface normal. Diffraction patterns were obtained by propagating a green laser (543.5 nm) through the samples. The distance between the sample and the projection plane was ≈100 cm for recording transmitted diffraction patterns of folding systems and ≈19 cm for reflected diffraction patterns of SIO or superstructure systems. All measurements were performed in ambient conditions.

### Device Design for Multi‐Directional and Multimodule Imaging

1) *World Architecture*: First, a 3‐second exposure to water vapor was utilized to create a “world architecture” pattern on a large‐scale SIO film measuring 12 cm × 3 cm. Next, three pieces of fold‐free Silk/PDMS bilayers were sculpted into different world's famous architectural patterns, and templated folding surfaces with orientations parallel, perpendicular, or inclined (at a 45° angle) to the direction of the architectures were created through unidirectional stretching and releasing. Lastly, the display system was assembled by piecing the three folding systems together onto the patterned SIO with the aid of a thin adhesion layer.
2) *Twelve Chinese Zodiacs*: First, twelve pieces of SIO films measuring 3 cm × 2.5 cm were patterned into oracle bone inscriptions of twelve Chinese zodiacs, respectively, using water vapor exposure. Next, twelve pieces of fold‐free Silk/PDMS bilayers were locally etched into the animal patterns corresponding to the zodiacs, and unidirectional stretching/releasing was used to form templated folding surfaces. Similarly, twelve additional pieces of folding surfaces were fashioned into corresponding terrestrial branches. To create each displaying module, the folding systems with animal and terrestrial branch patterns were orthogonally superimposed onto the corresponding patterned SIO film. Finally, the “modularized” display system was assembled by arranging the modules into a 4 × 3 array.
3) *Leaf‐Shaped Camouflage Device*: First, four pieces of SIOs were post‐treated with UV light (1 h) to create patterns of leaf veins. Next, three pieces of fold‐free Silk/PDMS bilayers were locally etched into baron caterpillar, leave insect, and butterfly patterns, and templated folding surfaces were formed after unidirectional stretching and releasing. Additionally, multi‐directionally stretched folding bilayers were created, with a stretching ratio of 20%, to provide quasi‐isotropic light diffusion. All the SIOs and folding bilayers were reshaped to a leaf‐shaped geometry by laser cutting. The camouflage system was finally constructed by sequentially attaching a multi‐directionally stretched folding bilayer and a templated folding (or fold‐free) bilayer to the patterned SIO. These artificial leaves were intentionally positioned at different angles to ensure that the folding pattern of each design was individually visible.

### Viewable Angle (θ) Calculation

The viewable angle (*θ*) of the system to the observer can be calculated by leveraging the reflected light diffraction and is given by:

(1) $$\theta =\mathrm{ta}n^{-1}\frac{r}{d}$$

where *r* is the major or minor radius of the diffraction ellipse pattern, and *d* represents the distance between the sample and the projection plane.

### Numerical Simulation

To numerically reveal the diffuse transmission capacity of the folding, far‐field simulations were performed by employing the finite element method. The width, height, and thickness of the “Ω” shaped folding in the simulation were set to 500 nm, 800 nm, and 170 nm, respectively. The refractive index of the folding was set as 1.44. The light source was a plane wave with a wavelength of 550 nm and was incident at 0° with respect to the symmetric axis of the folding. Perfect matching layers were used to mimic an infinite space. The maxima for the mesh size of the folding and air domain were set to 50 and 80 nm, respectively.

## Conflict of Interest

The authors declare no conflict of interest.

## Author Contributions

Y.W. and T.W. conceived the idea and designed the research. T.W., Y.W., Y.F., C.J., and Y.‐E.J. performed research. Z.C. executed the optical simulations. All authors contributed to the data analysis. T.W., Y.W., and Y.L. wrote the manuscript. Y.W. and Y.L. supervised the research.

## Supporting information

Supporting Information

Supplemental Movie 1

Supplemental Movie 2

Supplemental Movie 3

Supplemental Movie 4

Supplemental Movie 5

## Data Availability

The data that support the findings of this study are available from the corresponding author upon reasonable request.
