# Supplementary material for: Angle‐Multiplexed 3D Photonic Superstructures with Multi‐Directional Switchable Structural Color for Information Transformation, Storage, and Encryption
Source: Adv Sci (Weinh). 2024 May 17;11(28):2400442. doi: 10.1002/advs.202400442 (PMC11267312; doi:10.1002/advs.202400442)
Supplement: Supplementary file 1 — Supporting Information [file ADVS-11-2400442-s005.docx]

Supporting Information

**Angle-multiplexed 3D photonic superstructures with multi-directional switchable structural color for information transformation, storage, and encryption**

*Tao Wang, Yu Wang*, Yinghao Fu, Zhaoxian Chen, Chang Jiang, Yue-E Ji, and Yanqing Lu**

**Supplementary Figures**


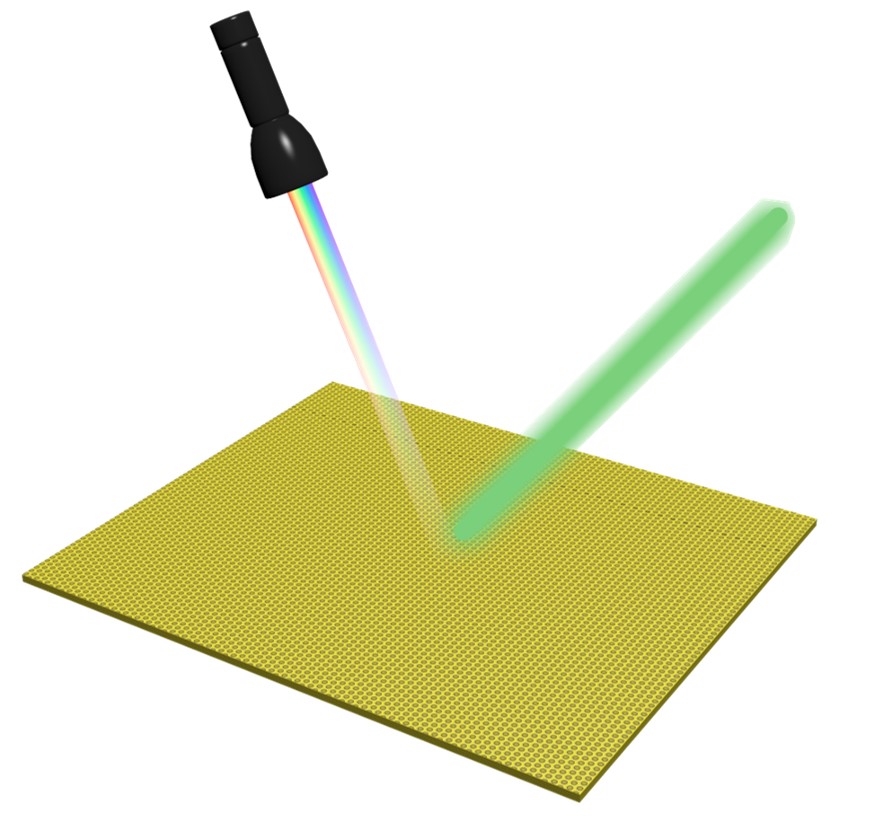


**Figure S1.** Schematic showing the specular light reflection from a conventional photonic crystal.


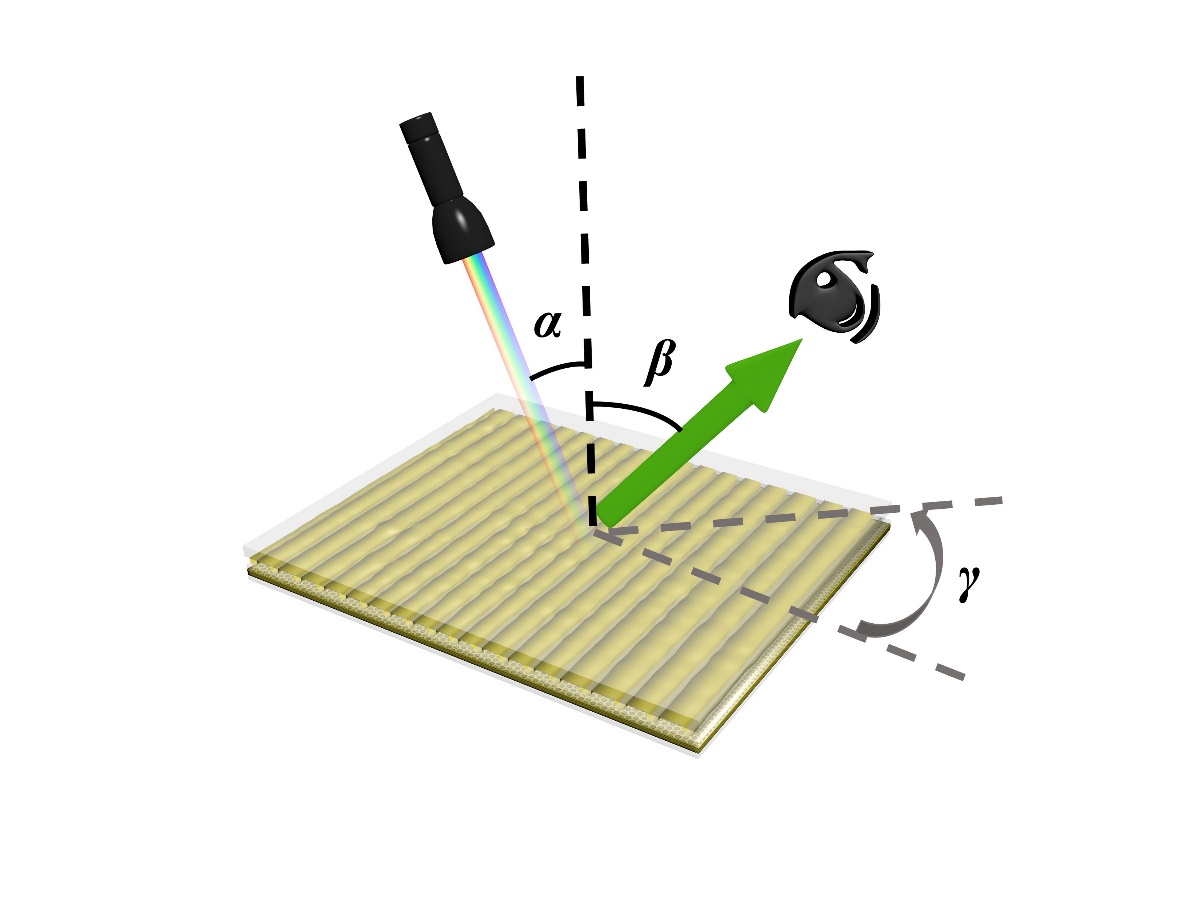


**Figure S2.** Schematic illustration of incidence angle (*α*), detection/viewing angle (*β*), and horizontal orientation angle (*γ*).


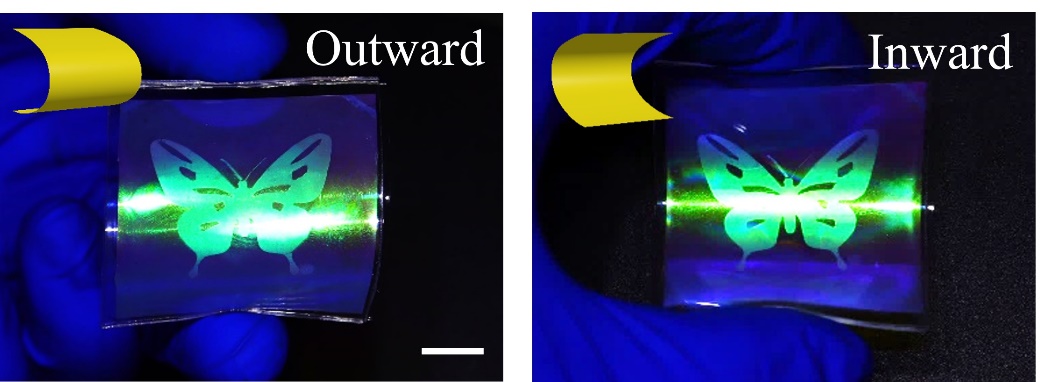


**Figure S3.** Photographs of the same photonic superstructure as in c under bending, showing a bright butterfly pattern regardless of whether it is bent inwards or outwards. Scale bars: 1 cm.


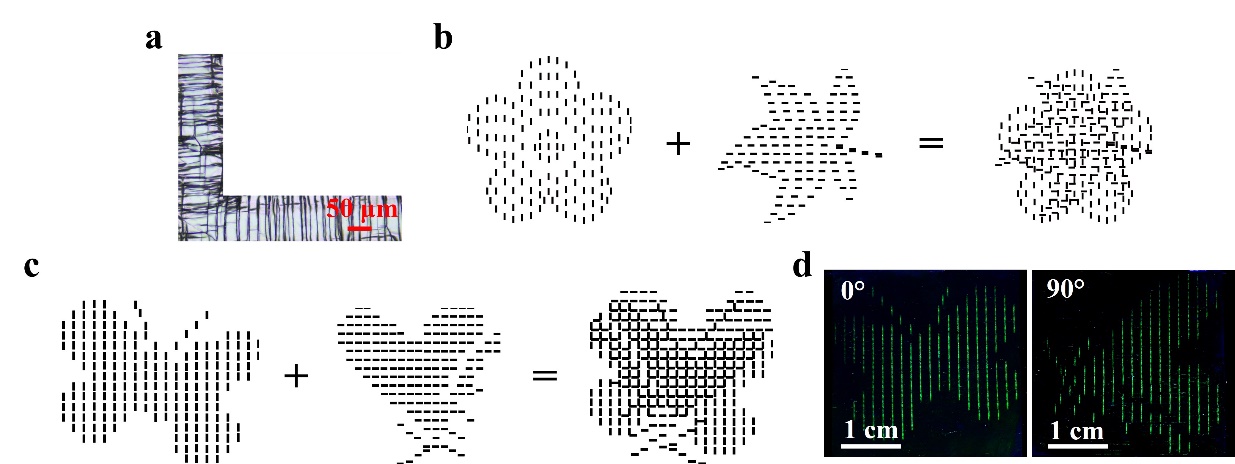


**Figure S4.** The generation of reversible pattern switching through creating orthogonal silk thin strips on the PDMS surface and applying biaxial stretching. (a) Microscopic image of two orthogonal silk thin strips (200 μm in width and 1 mm in length) after biaxial stretching, showing the formation of anisotropically aligned folding patterns in each strip with the direction perpendicular to the long axis. (b) The drawing designed to create a combination of sakura and maple leaf patterns. (c) The drawing designed to create a combination of butterflies in two forms. (d) Photographs showing the transition from a butterfly displaying open wings to a butterfly displaying closed wings with the increase of *γ* from 0° to 90° (*α* = 20°, *β* = 0°).


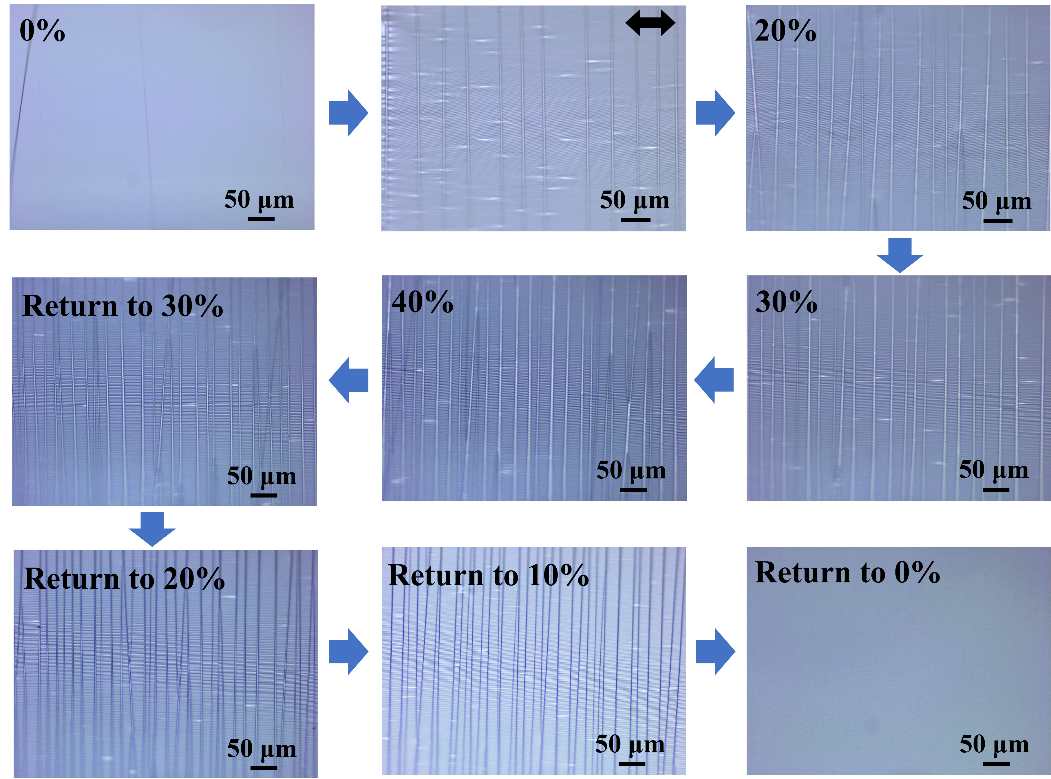


**Figure S5.** Optical microscopy images of the surface-oxidized PDMS under different levels of applied tensile strains. The film shows the formation of longitudinal cracks vertical to the strain and shallow transverse wrinkles perpendicular to the cracks during stretching. The number of quasi-periodically distributed cracks increases with the increase of applied strain. After releasing the tensile strain, the surface wrinkles become flattened and the cracks close up, yielding a completely flat surface again. The brittleness of the thin oxidized layer on top of the PDMS bulk generated by Oxygen plasma etching induces the formation of longitudinal cracks vertical to the stretching direction. The double-sided arrow indicates the stretching direction.


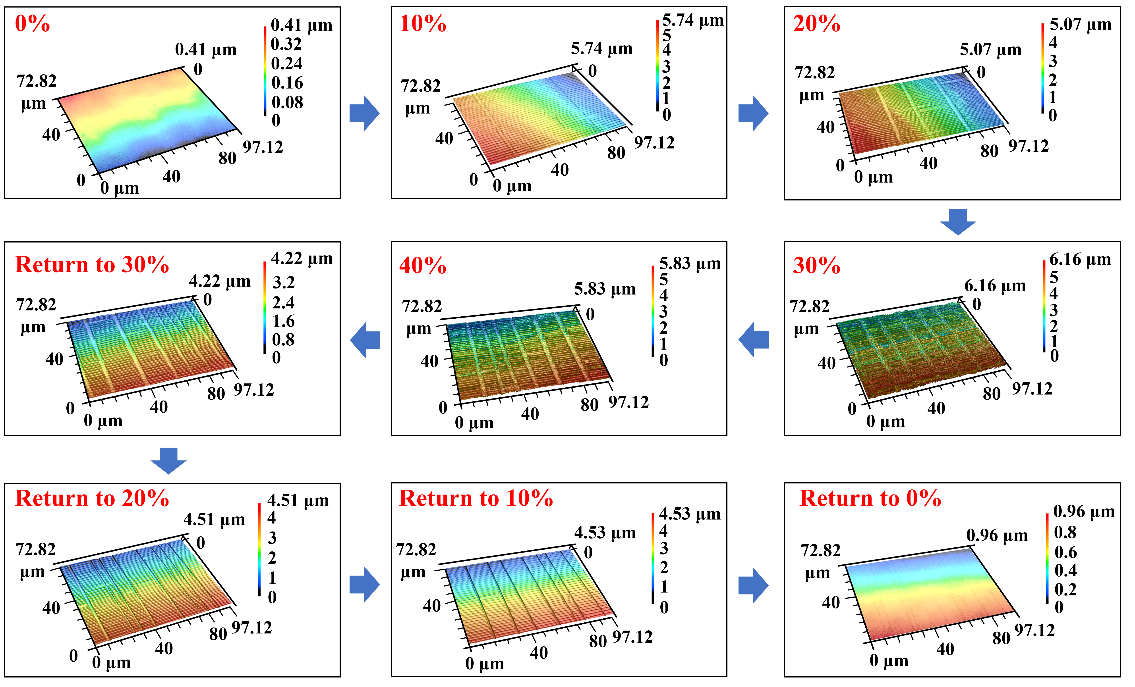


**Figure S6.** Laser-scanning confocal microscope (LSCM) images showing the surface topography of surface-oxidized PDMS under various applied tensile strains.


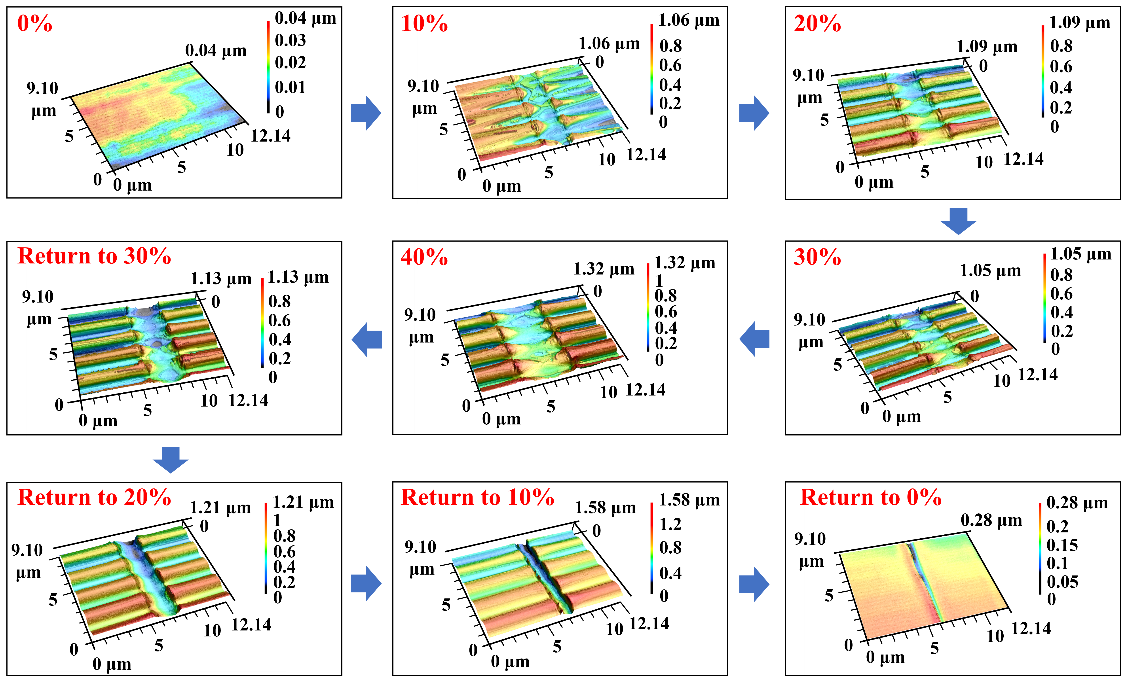


**Figure S7.** 3D LSCM images showing the evolution of the longitudinal crack and transverse wrinkles upon stretching and releasing within 40% strain in the surface-oxidized PDMS system.


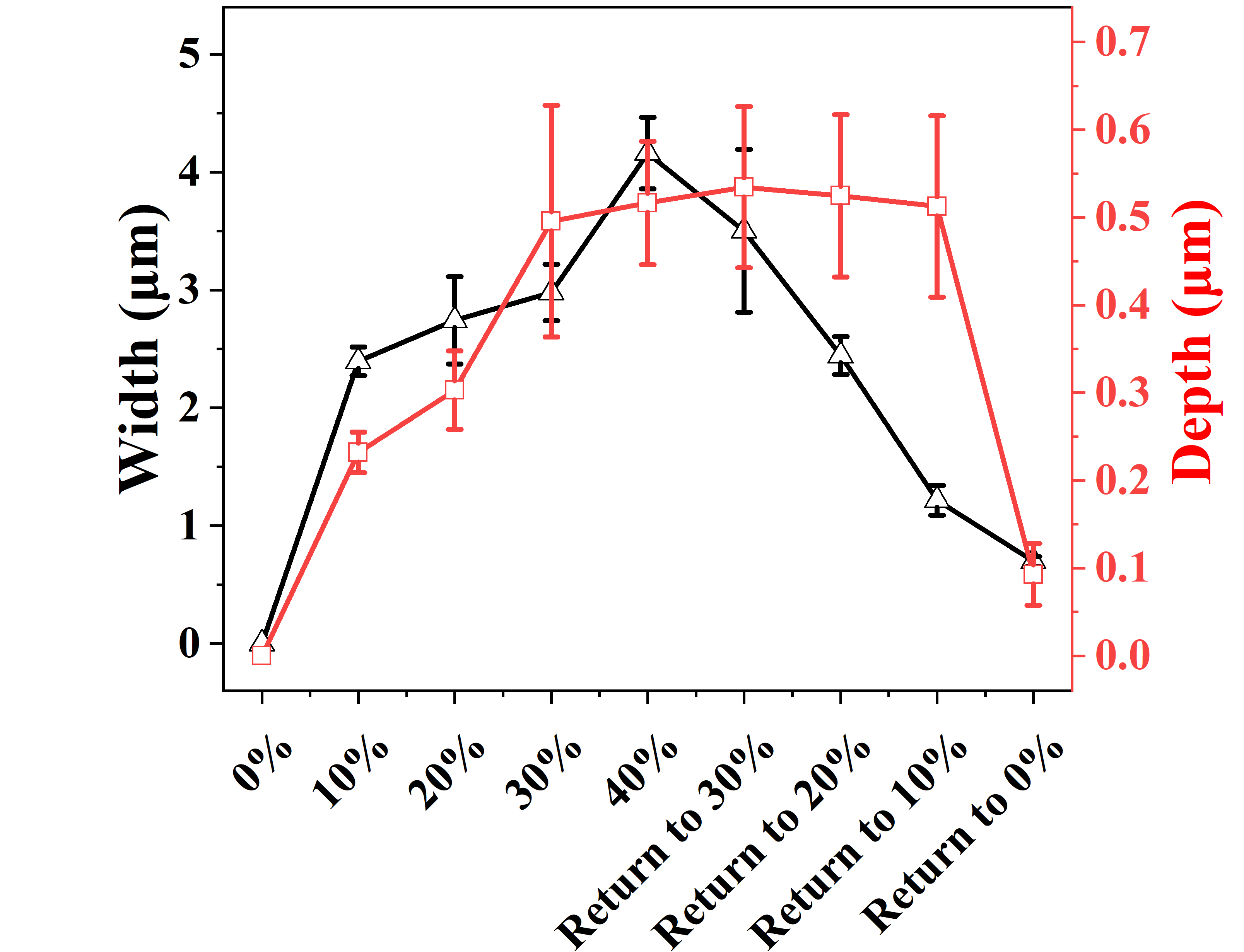


**Figure S8.** Dependence of crack width and depth on the applied strain upon stretching and releasing within 40% strain in the surface-oxidized PDMS system. The width of the crack increases with the increase of strain and gradually decreases during the stress-release process. The depth of the crack increases with increasing strain, but it remains relatively constant during the stress-release process and experiences a sharp decrease after the stress is completely released.


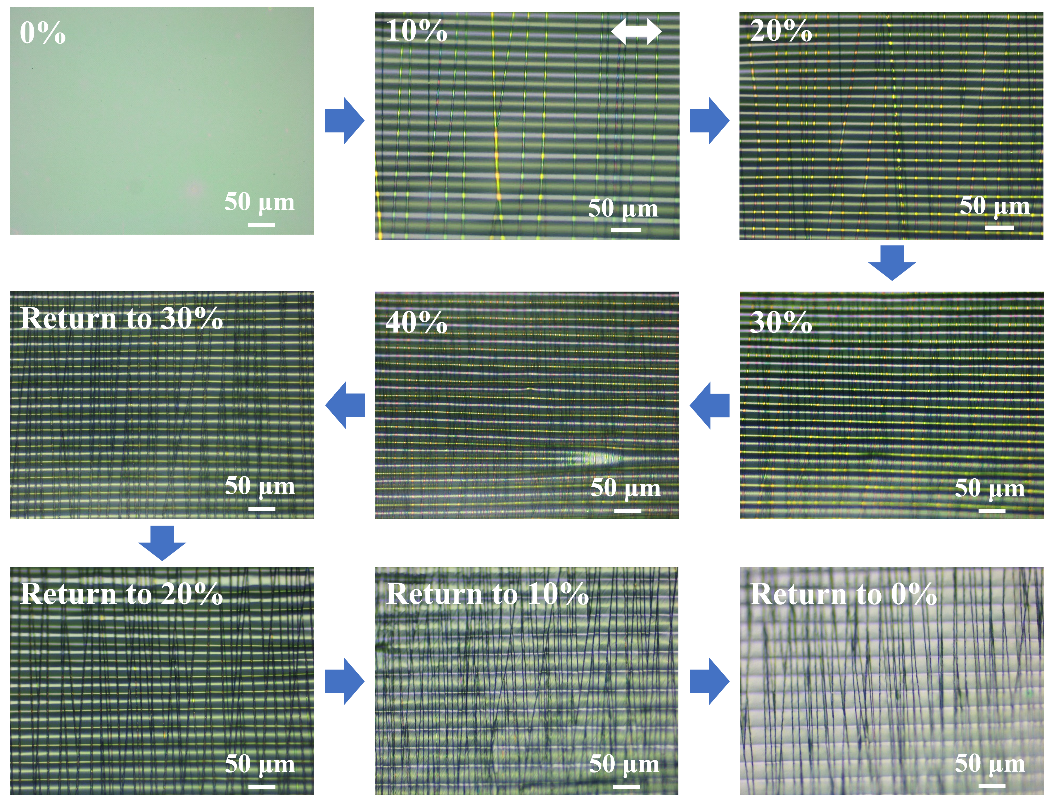


**Figure S9.** Optical microscopy images of the Silk/PDMS bilayer system under various applied strains. The number of quasi-periodically distributed cracks in the PDMS layer, which are oriented perpendicular to the stretching direction, increases with the increase of applied strain. The silk thin layer experiences plastic deformation during stretching, followed by subsequent folding upon release of the applied strain. During the stretching process, regular wrinkles form in parallel to the applied strain, and subsequently, upon release of the strain, they gradually decrease in amplitude, and lateral folds with low aspect ratio form along the wrinkle valleys. The double-sided arrow indicates the stretching direction.


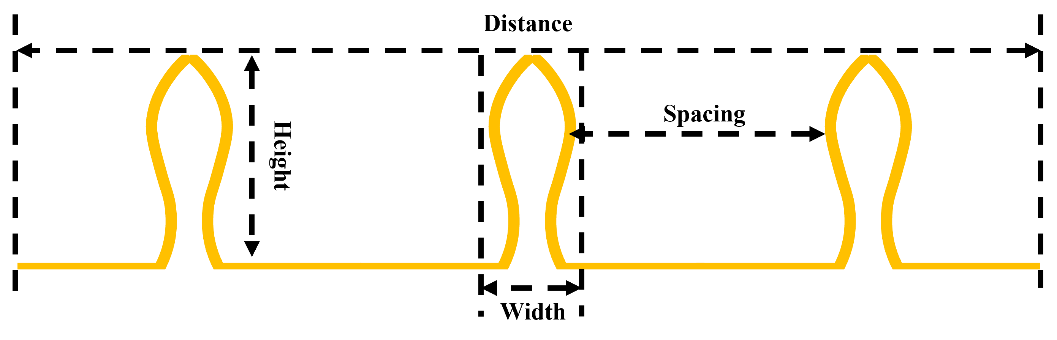


**Figure S10.** Schematic of the folding structure's height, distance, spacing, and width. The density of folds is given by: Density = Number/Distance, where Number is the number of folds included within the given distance.


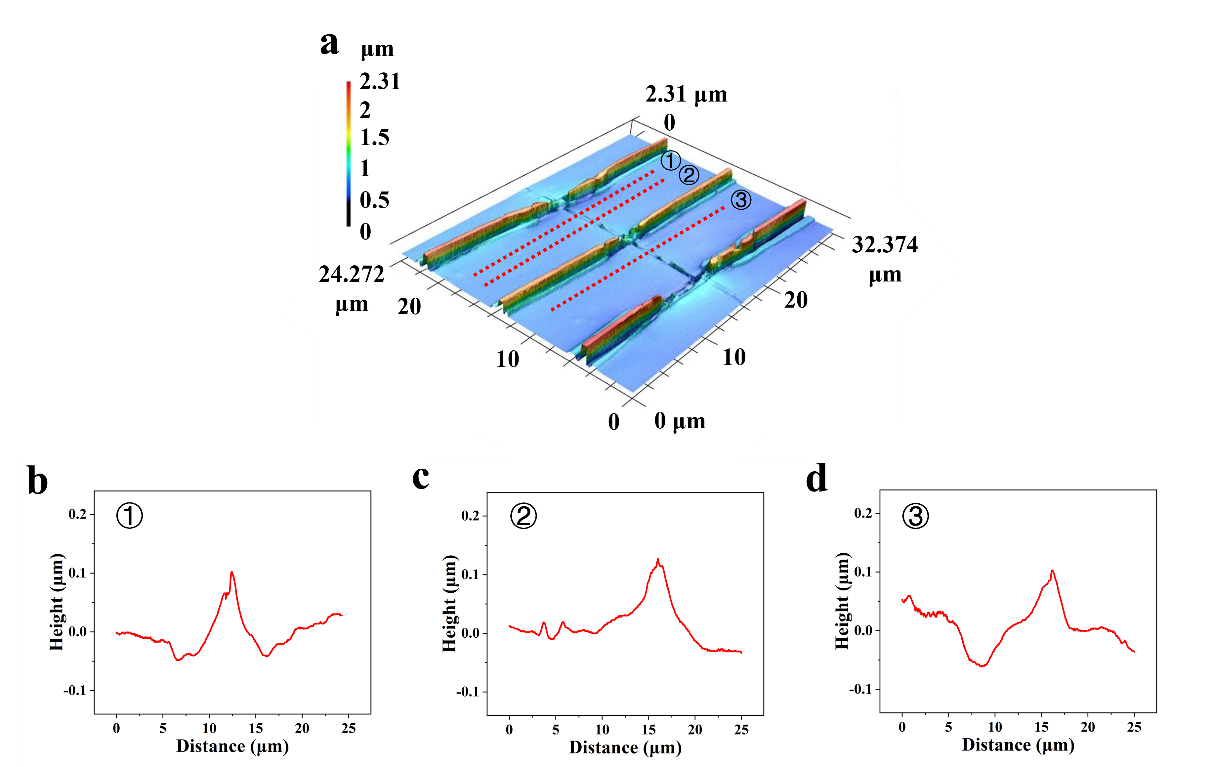


**Figure S11.** The formation of lateral folds after releasing the strain. (a) 3D LSCM image of a typical folding surface; (b-d) The cross-sectional profiles measured along the dashed line illustrated in a. The aspect ratio, defined as the height to the width of a folding structure, is calculated as 0.022 ± 0.04 (n = 3) based on curves shown in b-d.


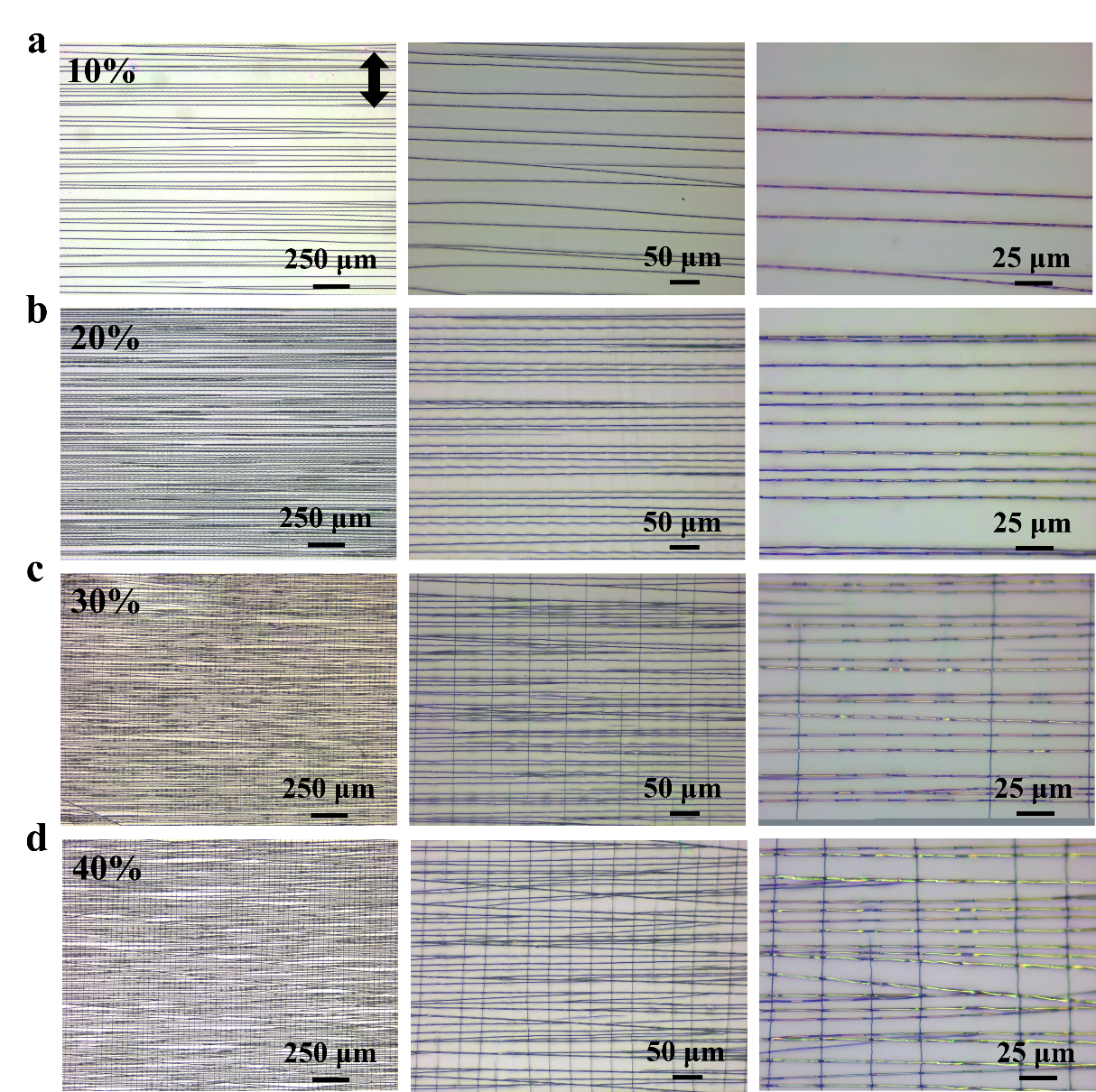


**Figure S12.** Optical microscopy images of the folding patterns formed under various strains. (a) 10% strain; (b) 20% strain; (c) 30% strain; (d) 40% strain. The arrow in a indicates the stretching direction.


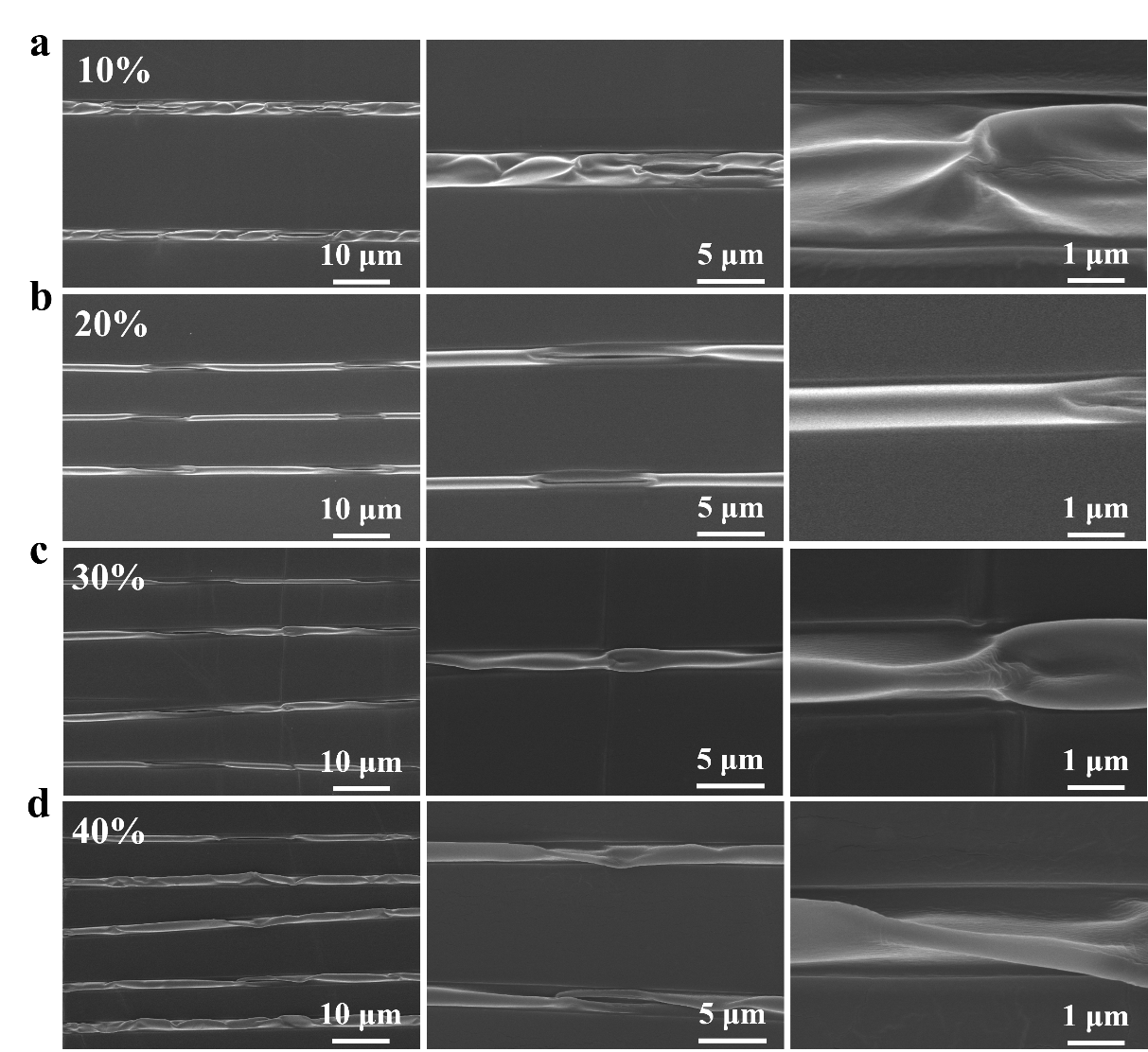


**Figure S13.** SEM images of the surface folding patterns formed under various strains. (a) 10%; (b) 20%; (c) 30%; (d) 40%.


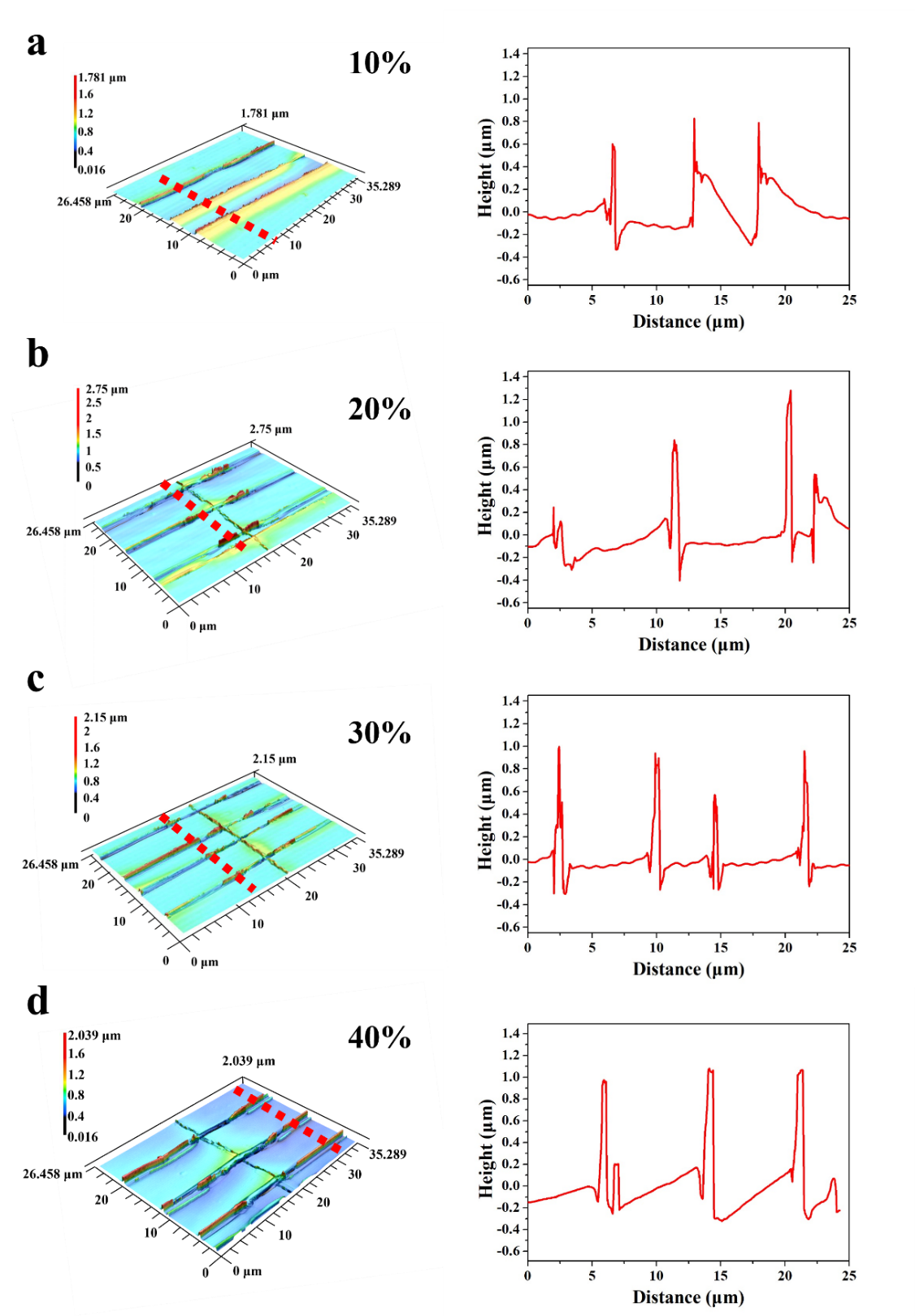


**Figure S14.** 3D LSCM images of the surface folding patterns formed under different strains. (a) 10%; (b) 20%; (c) 30%; (d) 40%. The cross-sectional profiles are measured along the dashed line illustrated in the corresponding LSCM images.


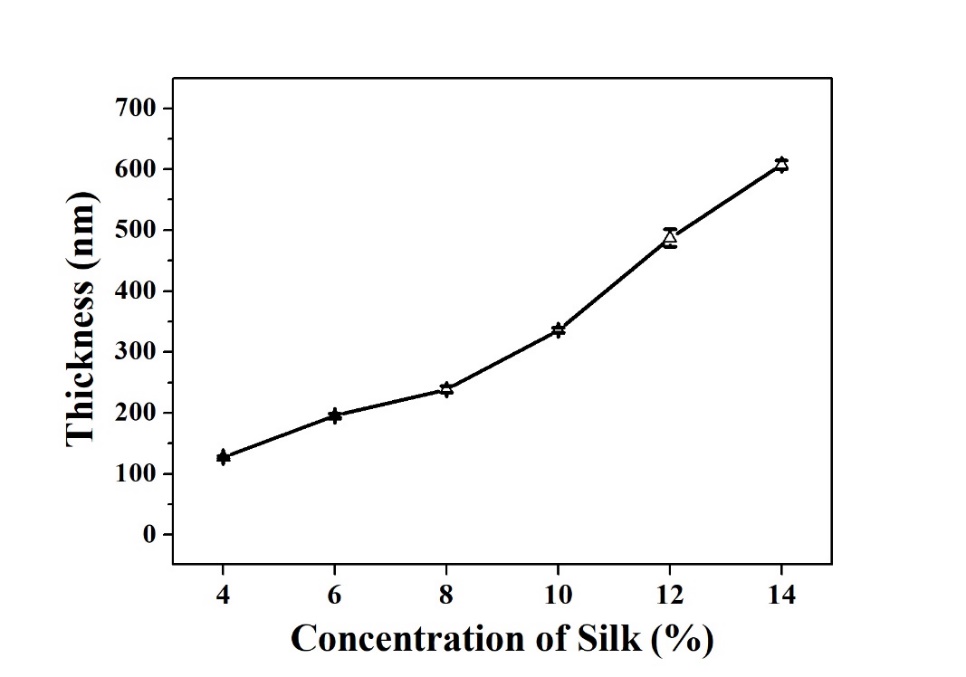


**Figure S15.** Dependence of the thickness of silk film on the concentration of silk fibroin.


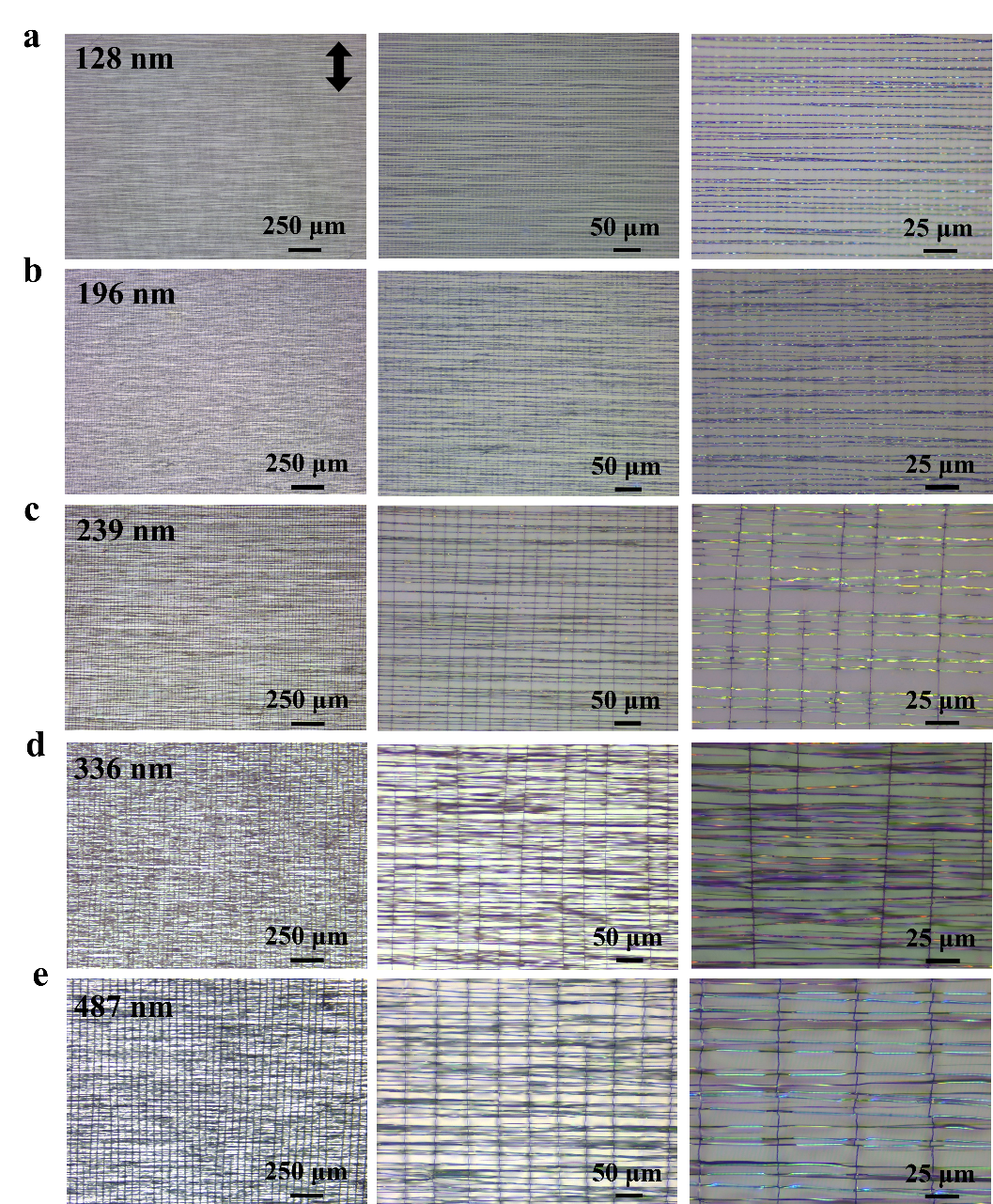


**Figure S16.** Optical microscopy images of the folding patterns with different thicknesses of silk film. (a) 128 nm; (b) 196 nm; (c) 239 nm; (d) 336 nm; (e) 487 nm. The arrow in (a) indicates the stretching direction.


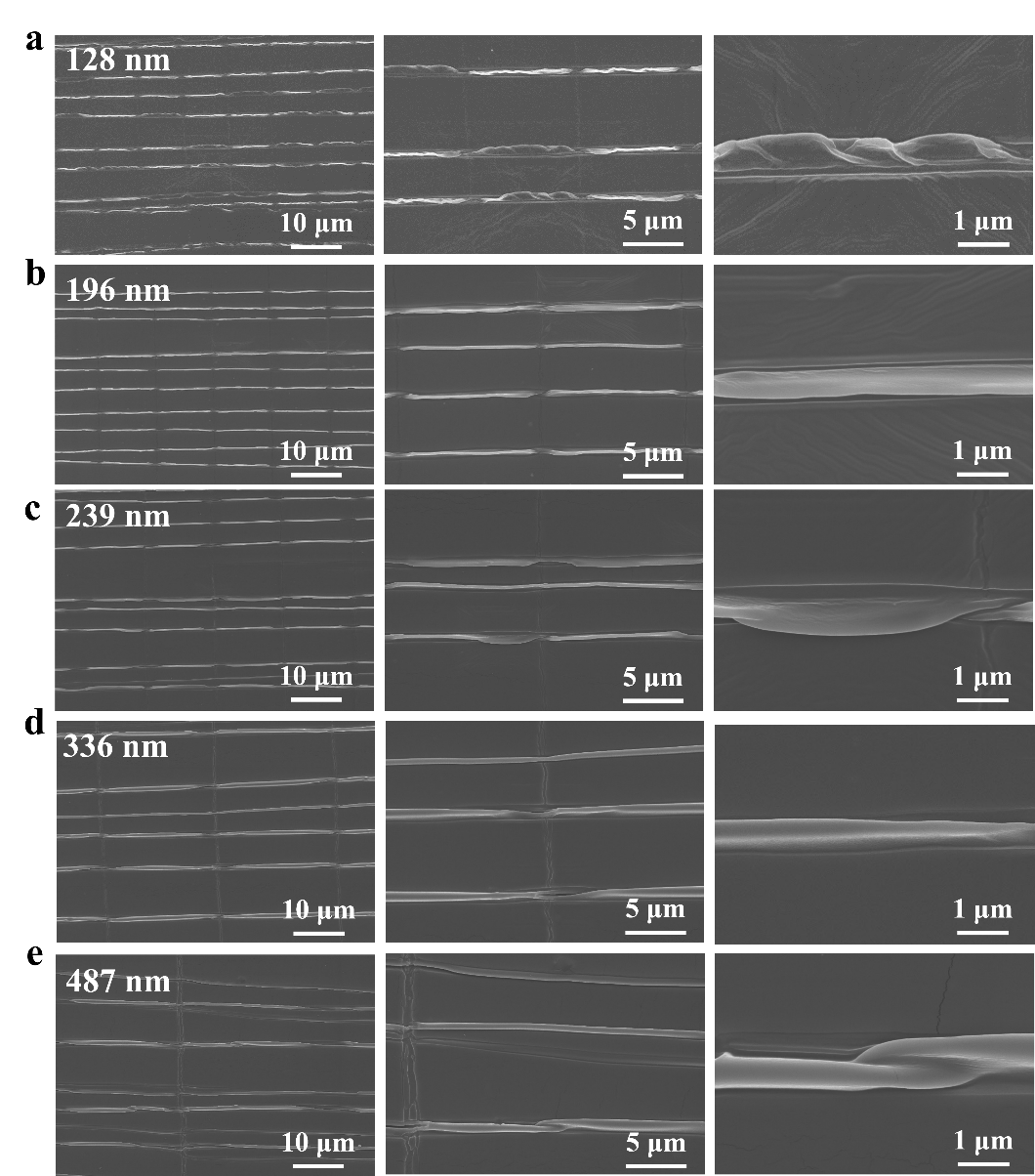


**Figure S17.** SEM images of the folding patterns with different thicknesses of silk film. (a) 128 nm; (b) 196 nm; (c) 239 nm; (d) 336 nm; (e) 487 nm.


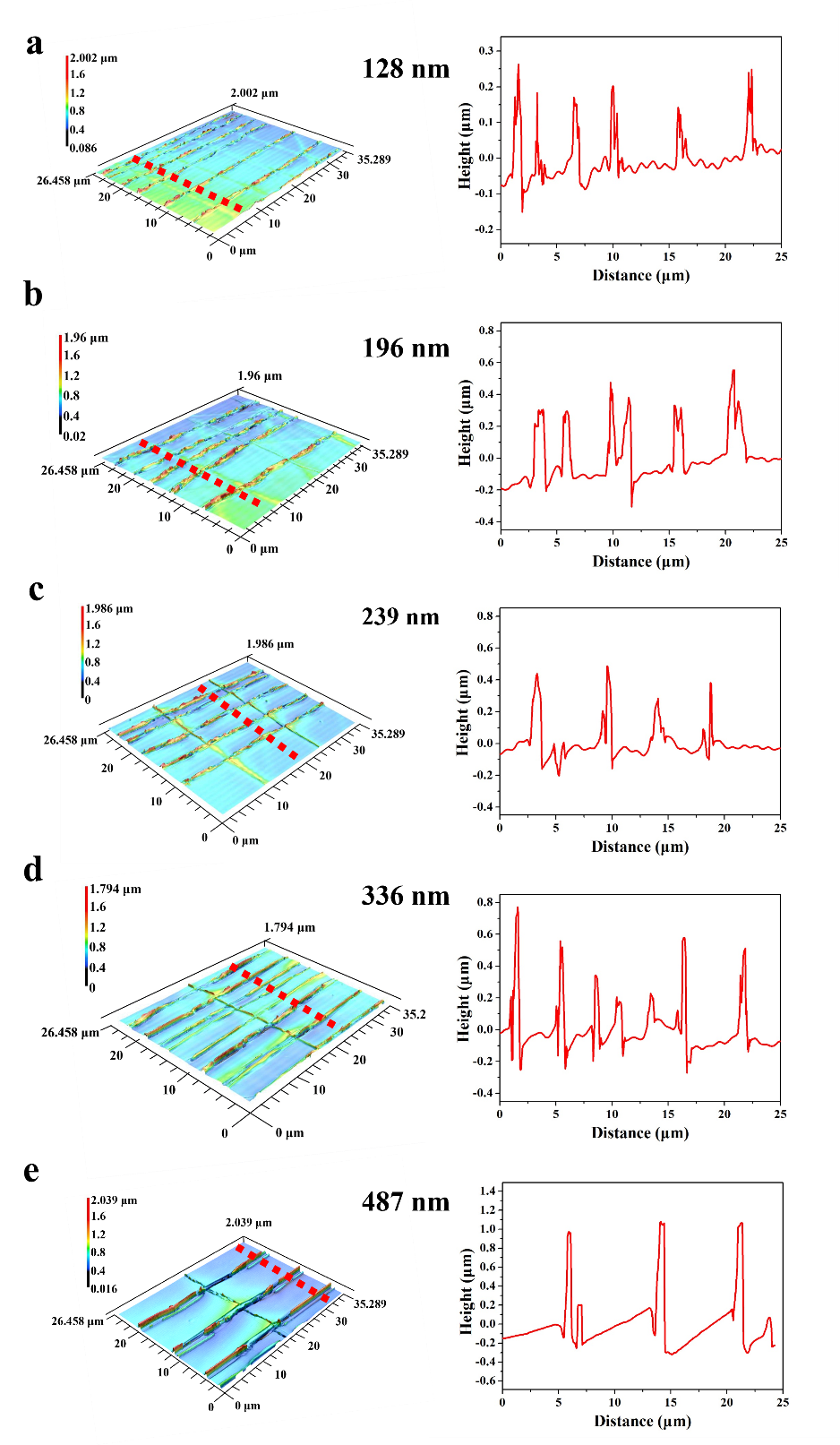


**Figure S18.**  3D LSCM images of the folding patterns with various thicknesses of silk film. (a) 128 nm; (b) 196 nm; (c) 239 nm; (d) 336 nm; (e) 487 nm. The cross-sectional profiles are measured along the dashed line illustrated in the corresponding LSCM images.


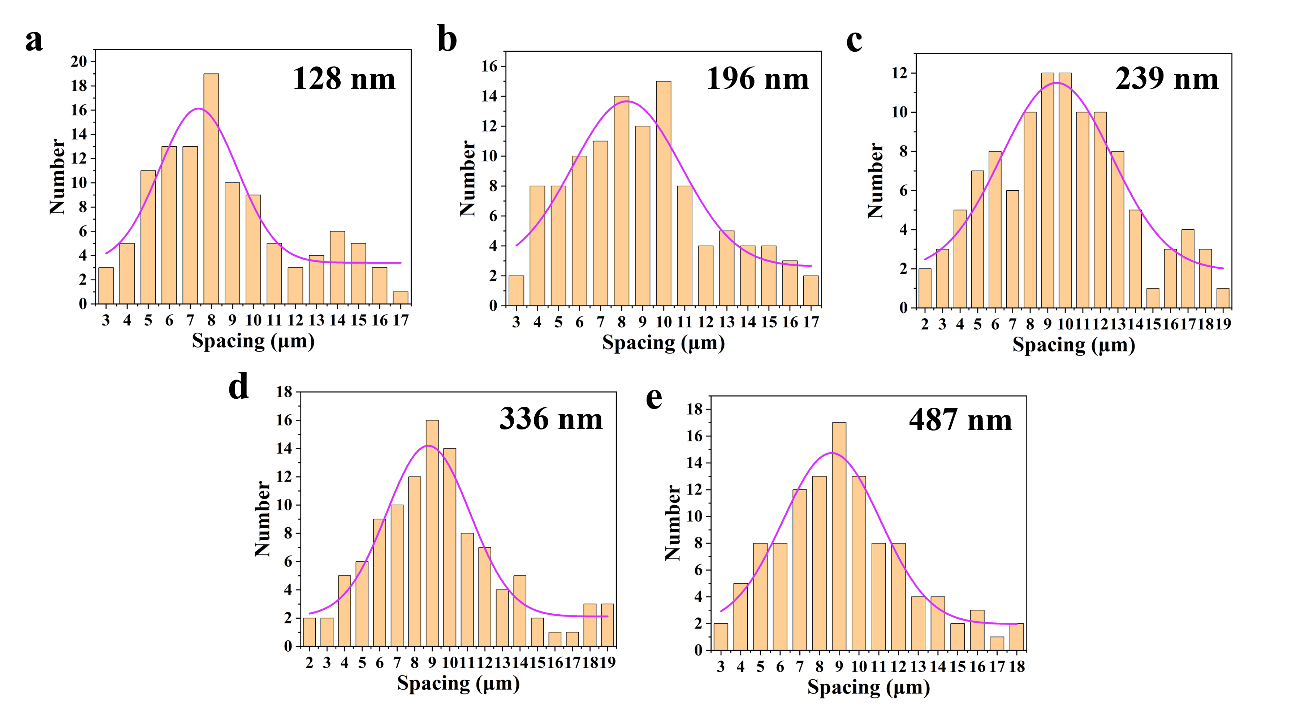


**Figure S19.** The size distribution of the spacing between two folds of the folding structure with various thicknesses of silk film. The pink line shows the Gaussian fit of the spacing size distribution.


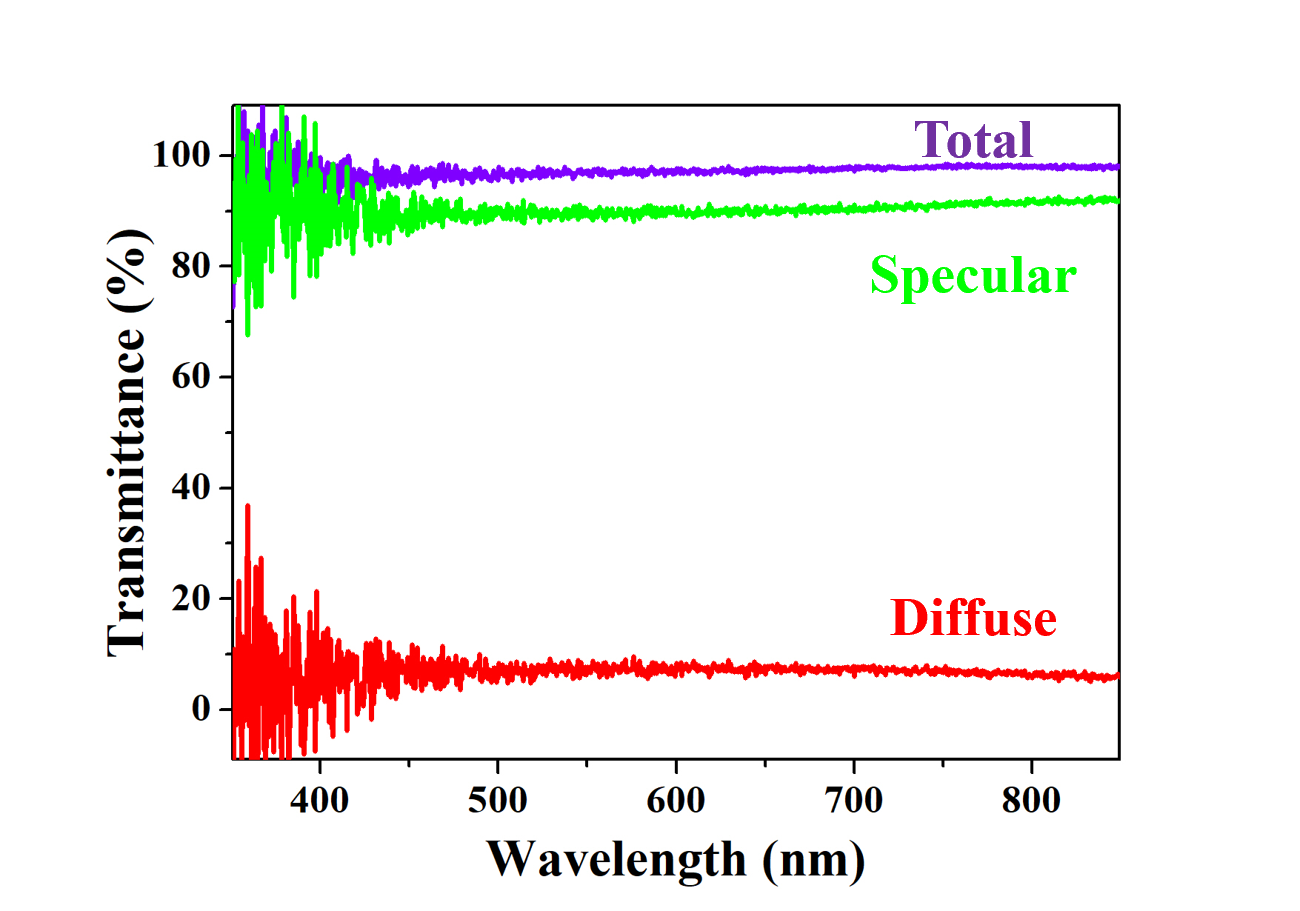


**Figure S20.** Total, specular, and diffuse transmittance spectra of the flat silk/PDMS bilayer system.


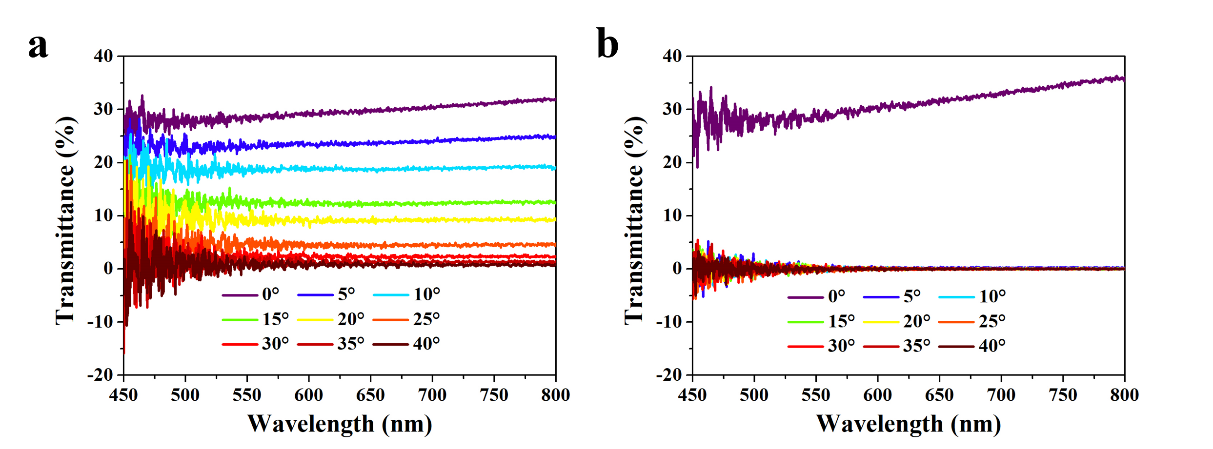


**Figure S21.** Transmission spectra of silk/PDMS anisotropic folding system under diffusive transmission mode with detection angle (*β*) switching from 0° to 40°. (a) *α* = 0°, *γ* = 0°; (b) *α* = 0°, *γ* = 90°.


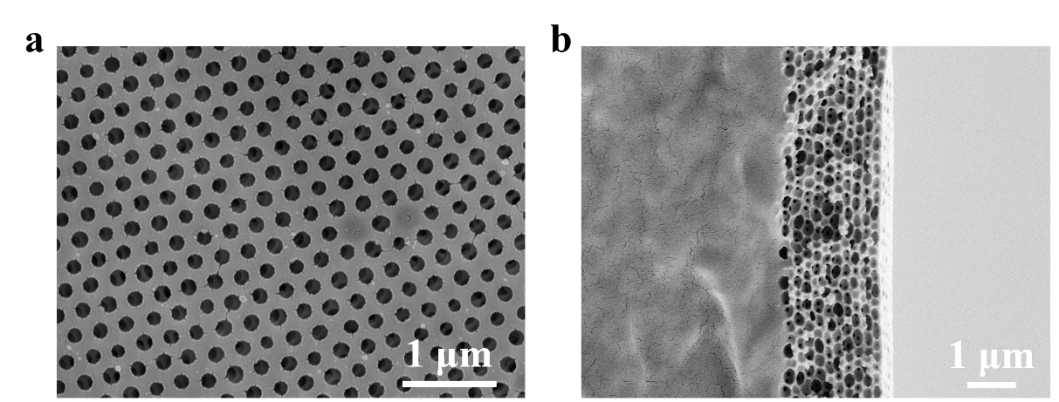


**Figure S22.** Microstructures of a 10-layer SIO created using colloidal crystal multilayers made of polystyrene spheres with a diameter of 300 nm as a template. (a) Surface SEM image showing the ordered hexagonal arrays of air cavities; (b) Cross-sectional SEM image displaying the ordered hollow structure with air holes on the wall.


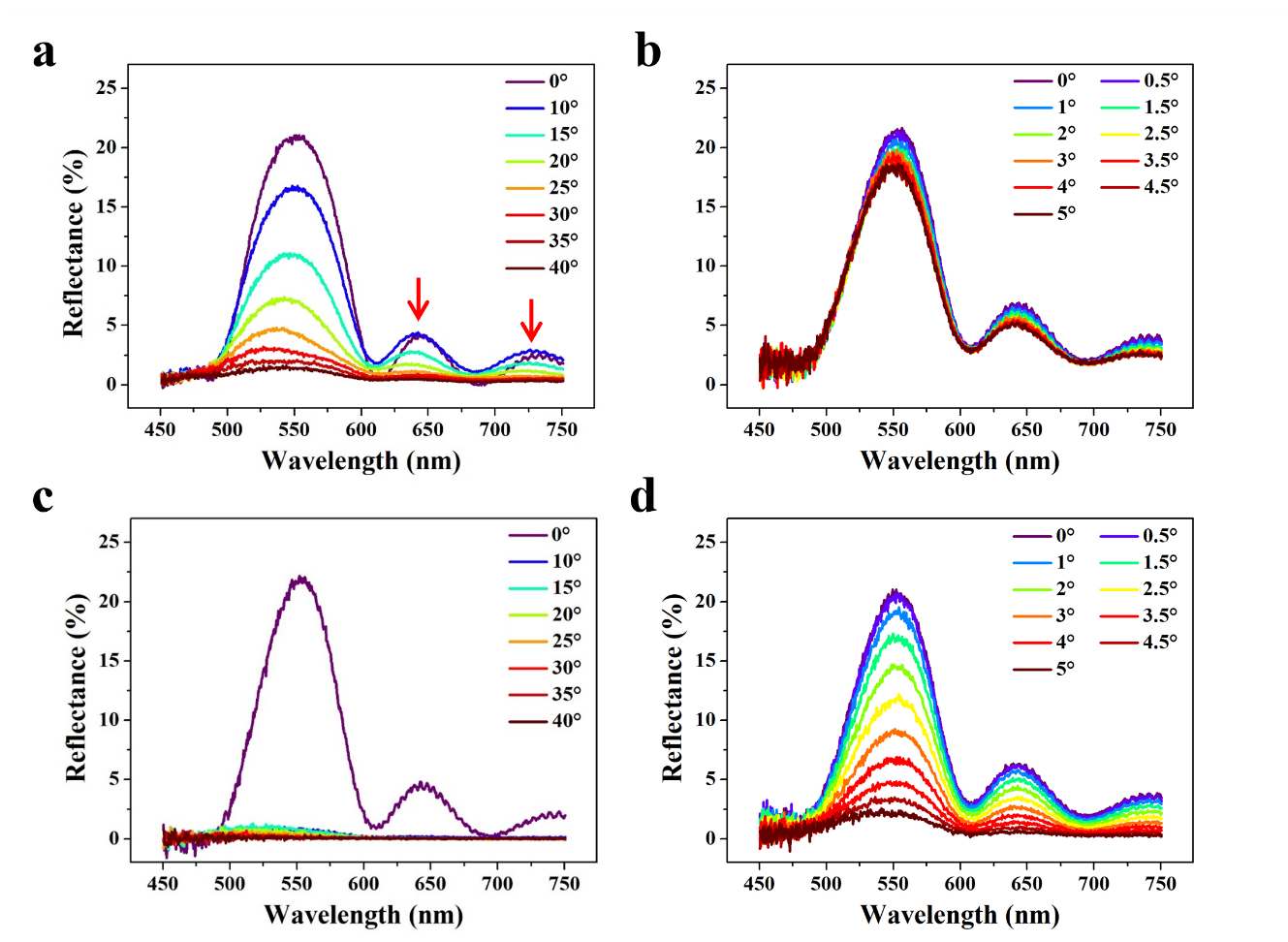


**Figure S23.** Reflectance spectra of the photonic superstructure measured under diffusive reflection mode with various detection angles (*β*). (a, b) *α* = 0°, *γ* = 0°; (c, d) *α* = 0°, *γ* = 90°. The reflectance peak centered at ~550 nm is assigned to the stop-band of the inverse opal, while the small peaks at longer wavelengths (indicated by arrows) are attributed to the Fabry–Perot fringes that result from the reflections at the front and rear facets of the inverse opal.^[1]^


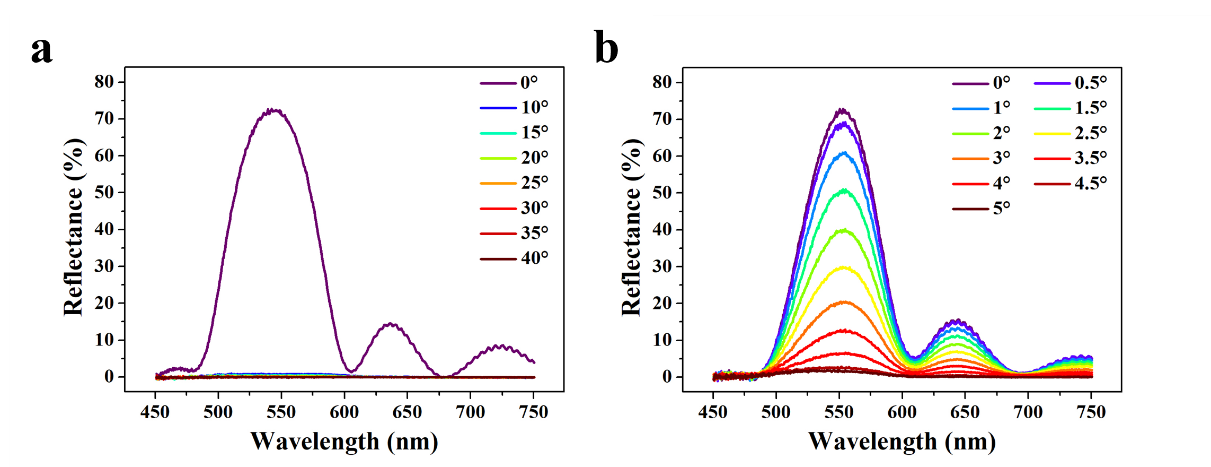


**Figure S24.** Reflectance spectra of silk inverse opal measured under diffusive reflection mode with various detection angles (*β*). (a) *α* = 0°, *β* = 0° ~ 40°; (b) *α* = 0°, *β* = 0° ~ 5°.


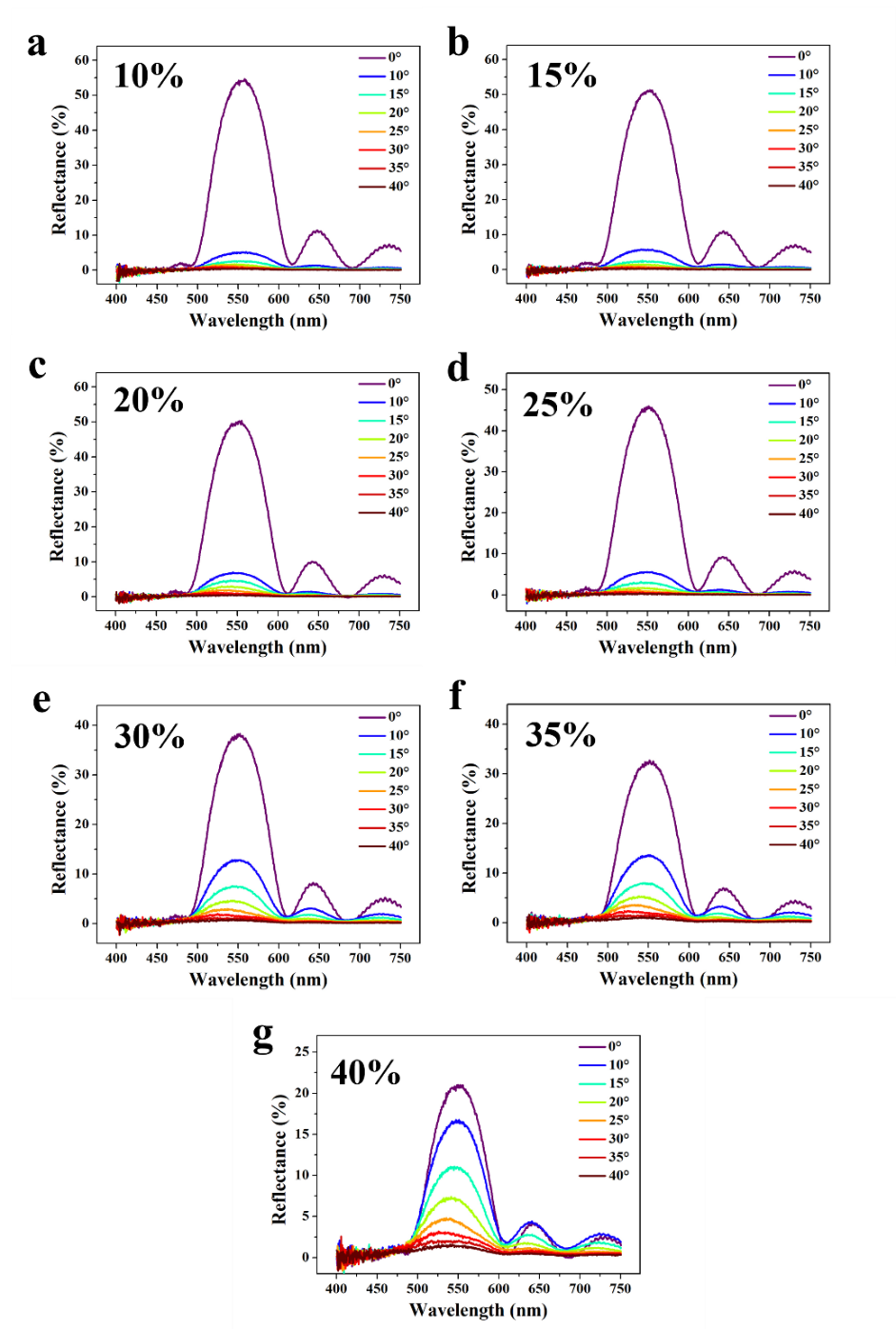


**Figure S25.** Reflectance spectra of the photonic superstructures with the folding structures being formed under different tensile strains at various detecting angles (*β*) (*α* = 0°, *γ* = 0°). (a) 10%; (b) 15%; (c) 20%; (d) 25%; (e) 30%; (f) 35%; (g) 40%.


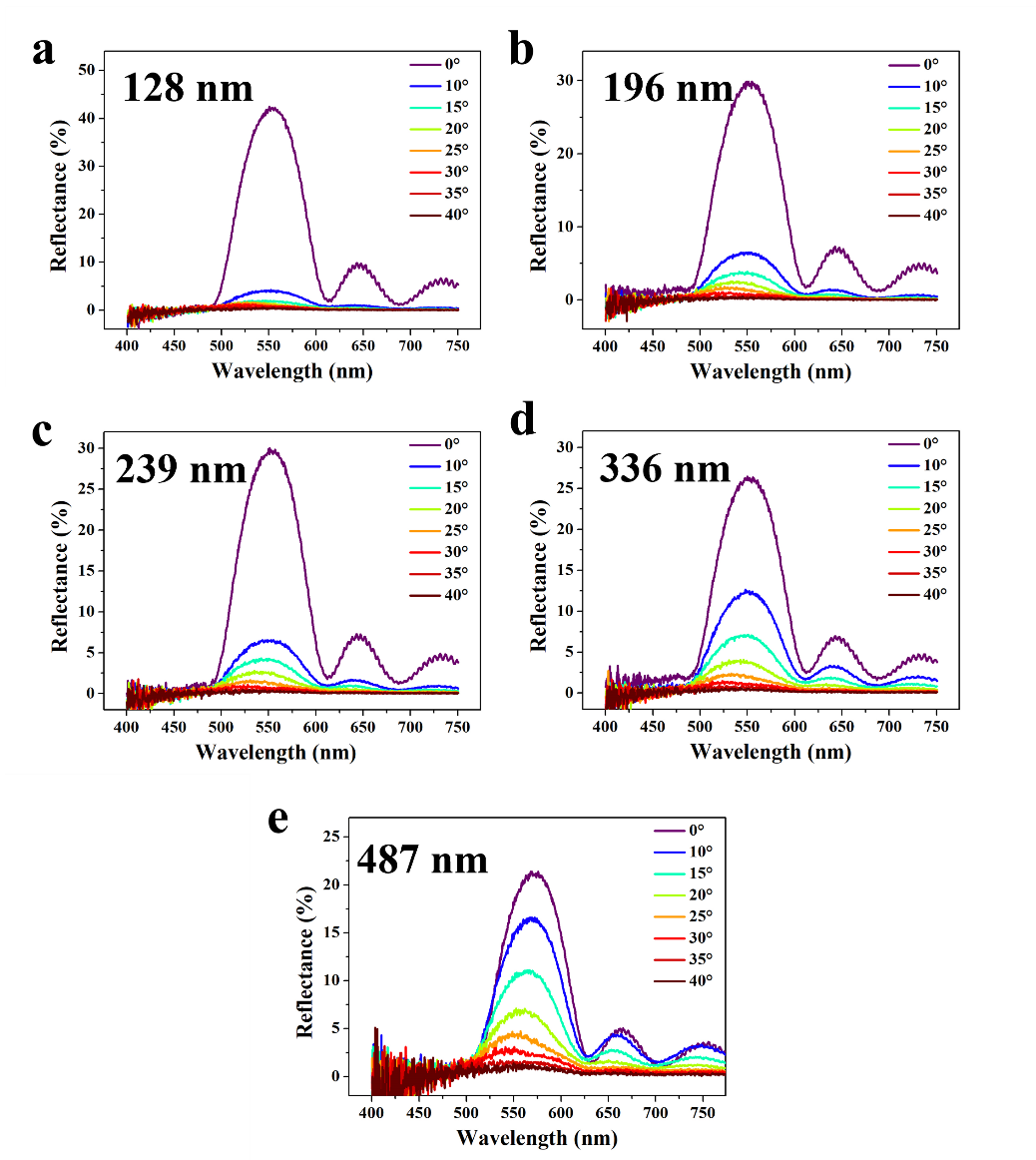


**Figure S26.** Reflectance spectra of the photonic superstructures with the folding structures being formed using different thicknesses of silk film at various detecting angles (*β*) (*α* = 0°, *γ* = 0°). (a) 128 nm; (b) 196 nm; (c) 239 nm; (d) 336 nm; (e) 487 nm.


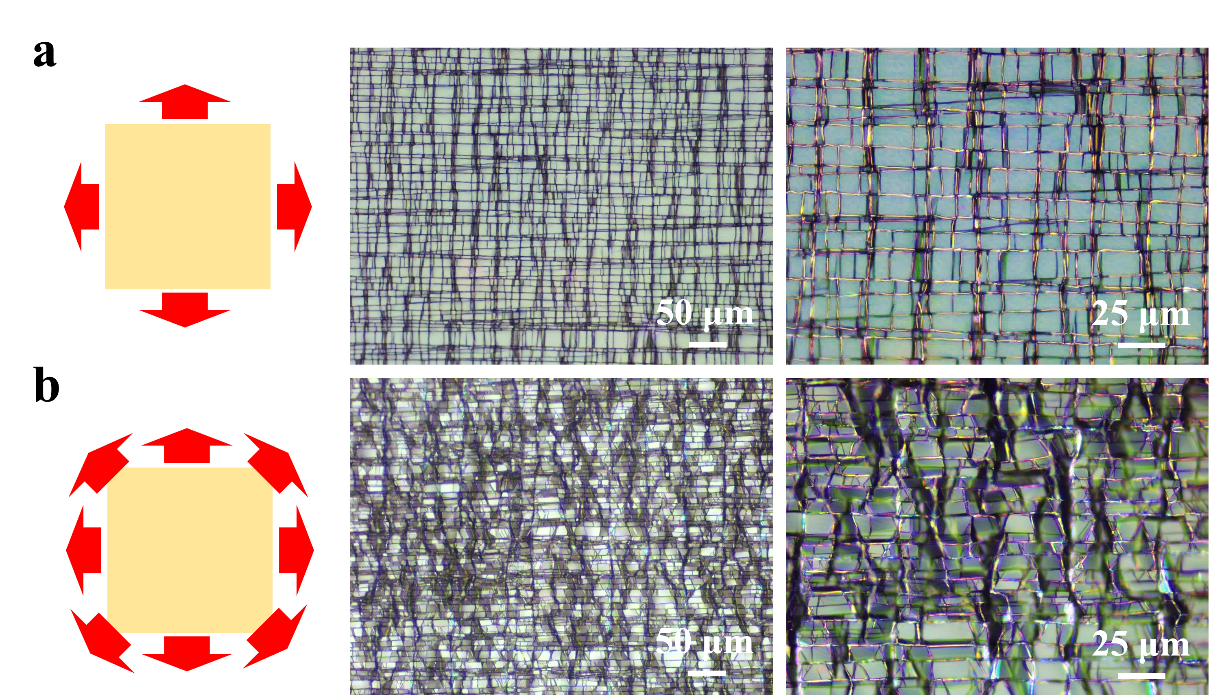


**Figure S27.** Surface morphologies of Silk/PDMS bilayers after biaxial stretching and multi-axis stretching. (a) Schematic of biaxial stretching mode and microscopic images of Silk/PDMS folding systems created by biaxial stretching. (b) Schematic of multi-axis stretching mode and microscopic images of Silk/PDMS folding systems created by multi-axis stretching.


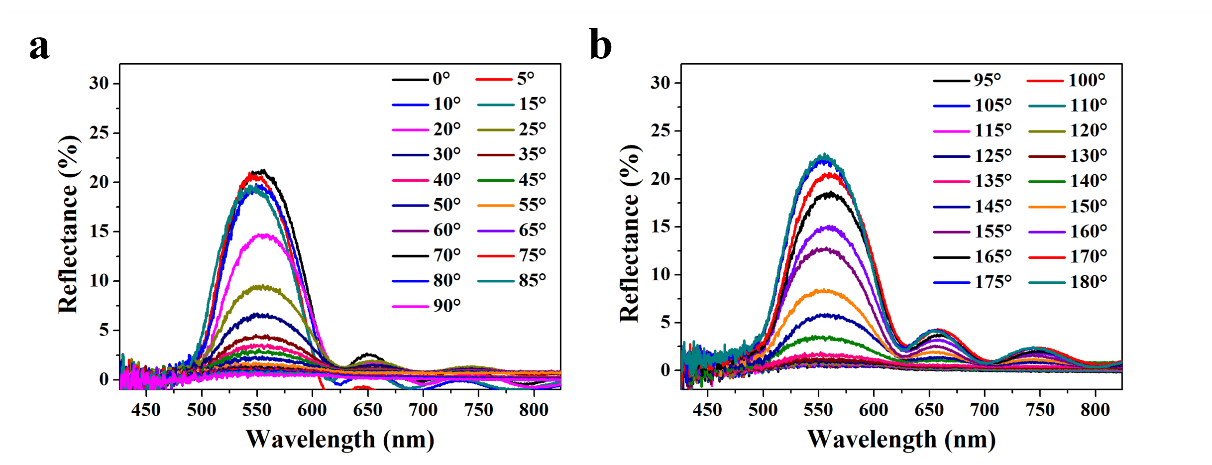


**Figure S28.** Optical properties of the photonic superstructure with anisotropically oriented folding structure at different horizontal orientation angles (*γ*). (a) *γ* = 0-90°; (b) *γ* = 95-180° (*α* = 0°, *β* = 10°).


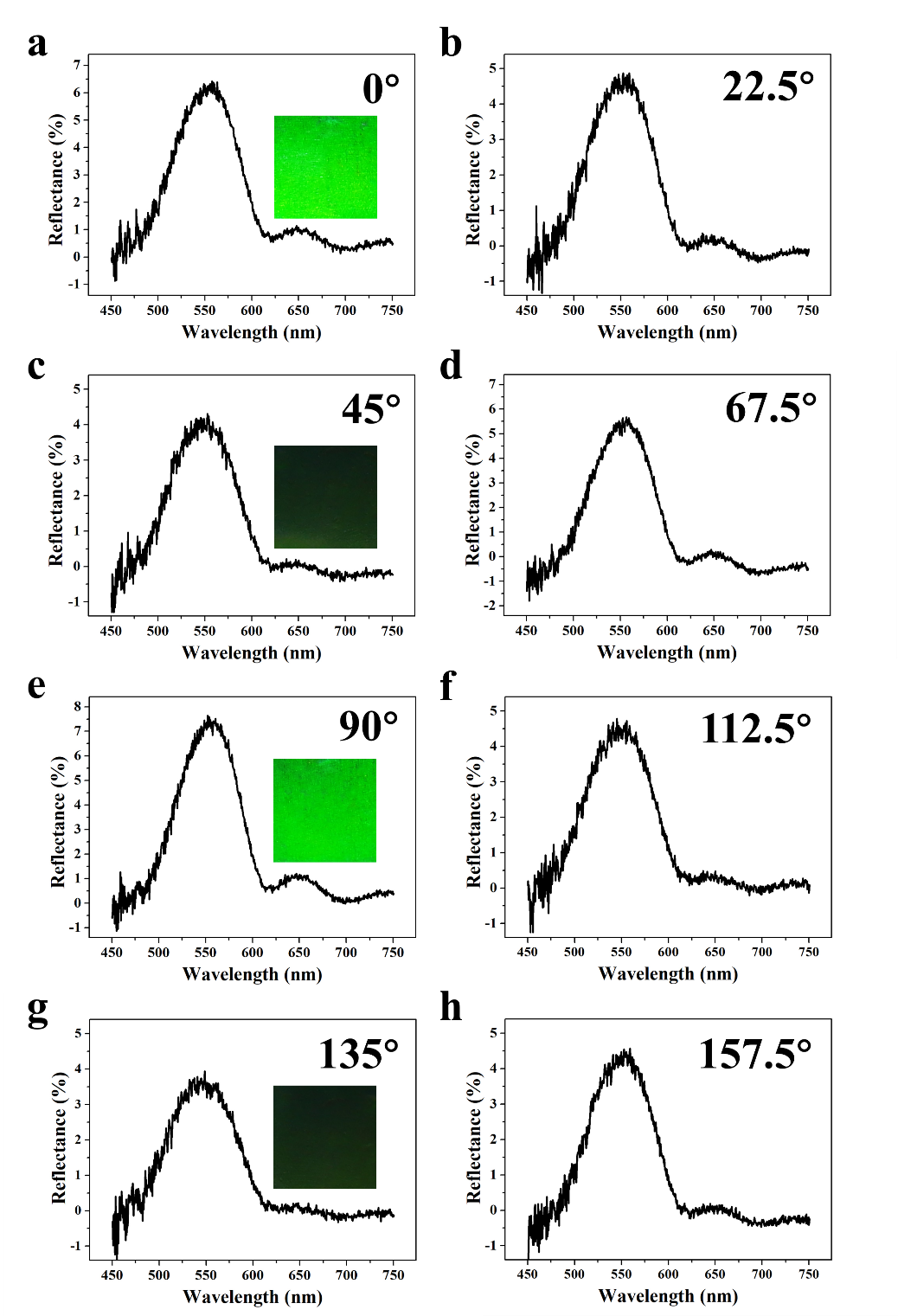


**Figure S29.** Optical properties of the photonic superstructure with biaxially stretched folding structure at different horizontal orientation angles (*γ*). (a) *γ* = 0°; (b) *γ* = 22.5°; (c) *γ* = 45°; (d) *γ* = 67.5°; (e) *γ* = 90°; (f) *γ* = 112.5°; (g) *γ* = 135°; (h) *γ* = 157.5°. Insets are the corresponding photographs of the sample. (*α* = 0°, *β* = 10°).


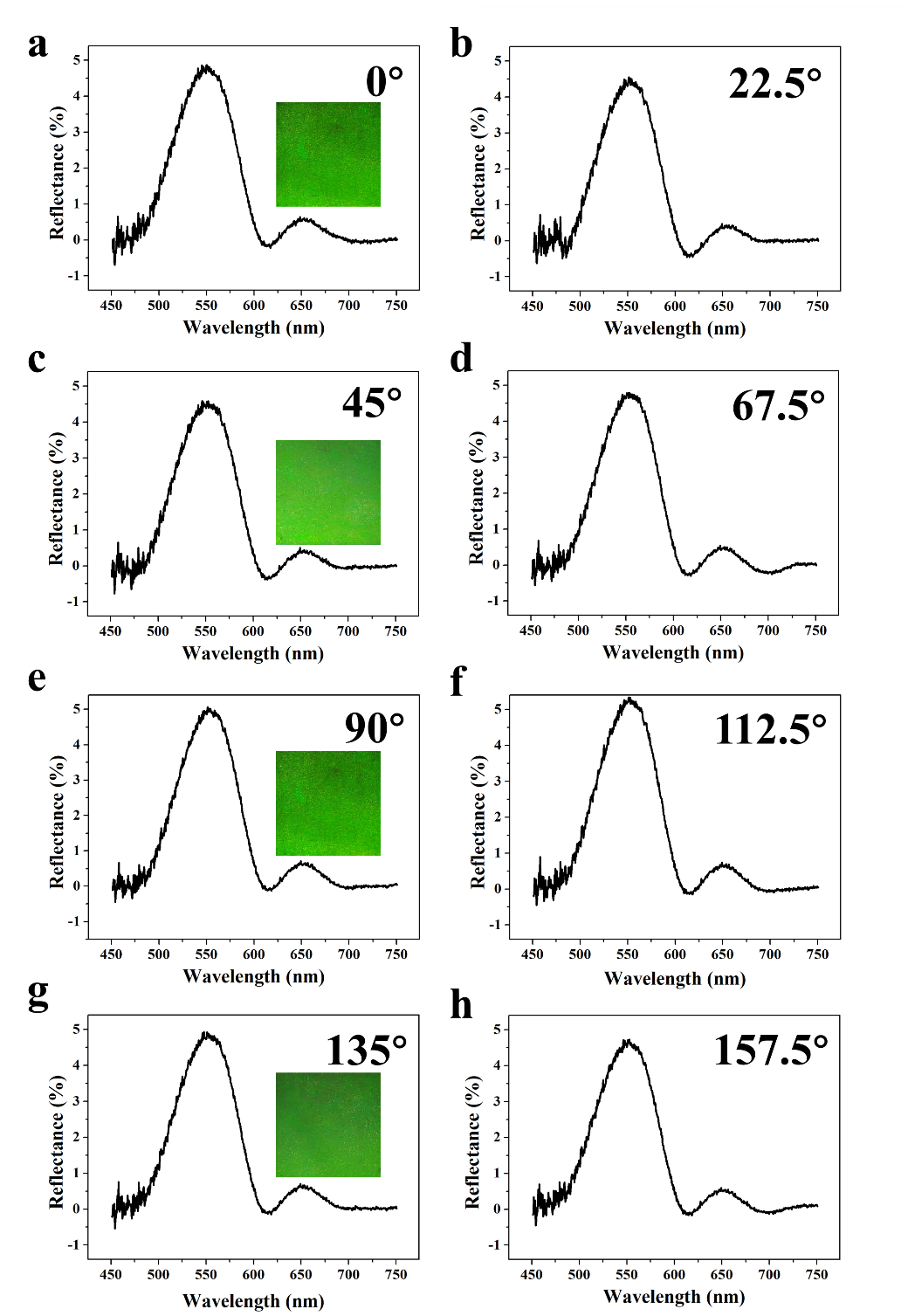


**Figure S30.** Optical properties of the photonic superstructure with randomly oriented folding structure (obtained through multi-axial stretching) at different horizontal orientation angles (*γ*). (a) *γ* = 0°; (b) *γ* = 22.5°; (c) *γ* = 45°; (d) *γ* = 67.5°; (e) *γ* = 90°; (f) *γ* = 112.5°; (g) *γ* = 135°; (h) *γ* = 157.5°. Insets are the corresponding photographs of the sample. (*α* = 0°, *β* = 10°).


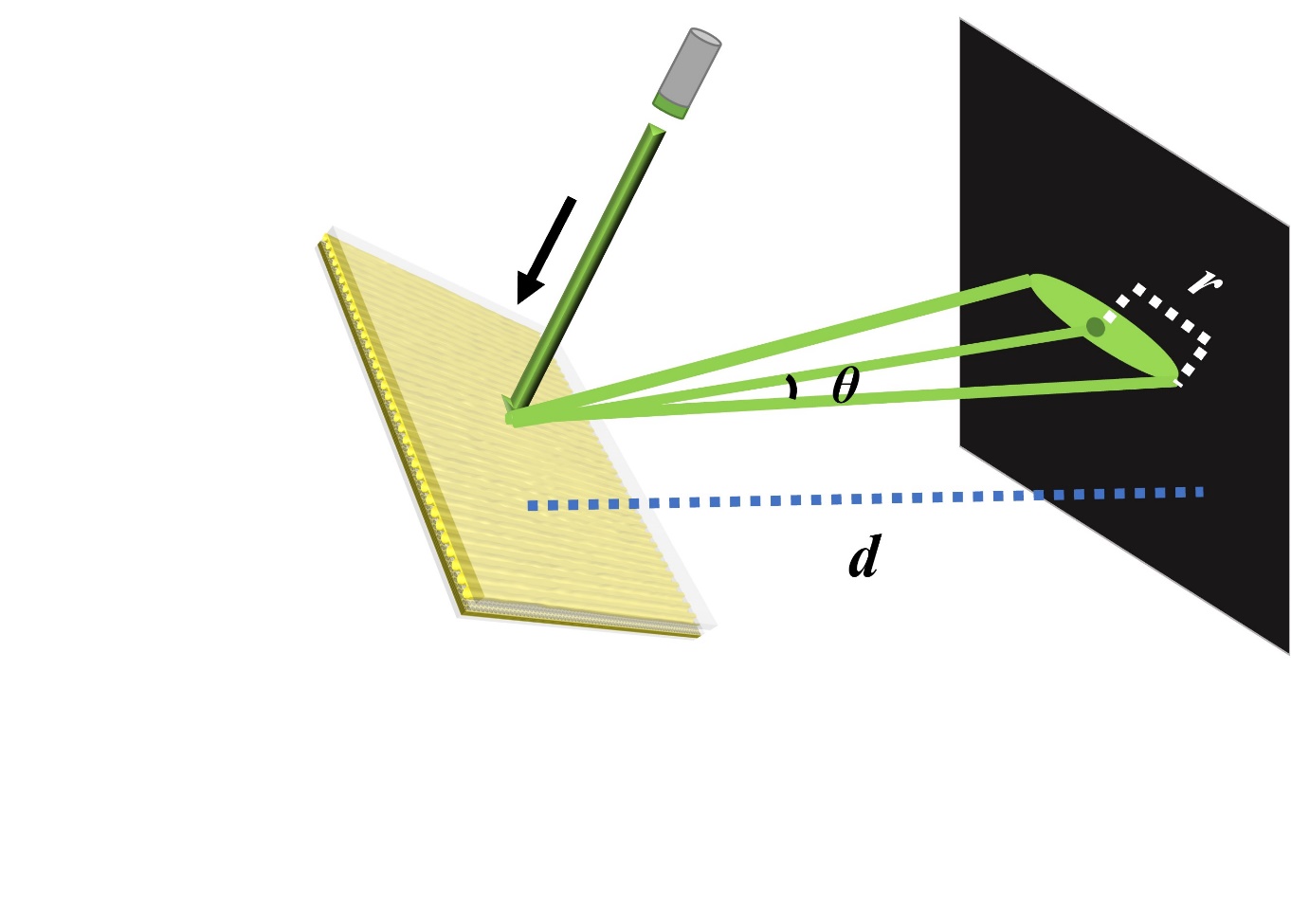


**Figure S31.** Schematic illustration of the spatial distribution of reflected light from the photonic superstructure. The viewing angle can be calculated by θ = arctan r/d. By calculation, r = 17, d = 19. As such, θ = 17/19 = 47.86°.


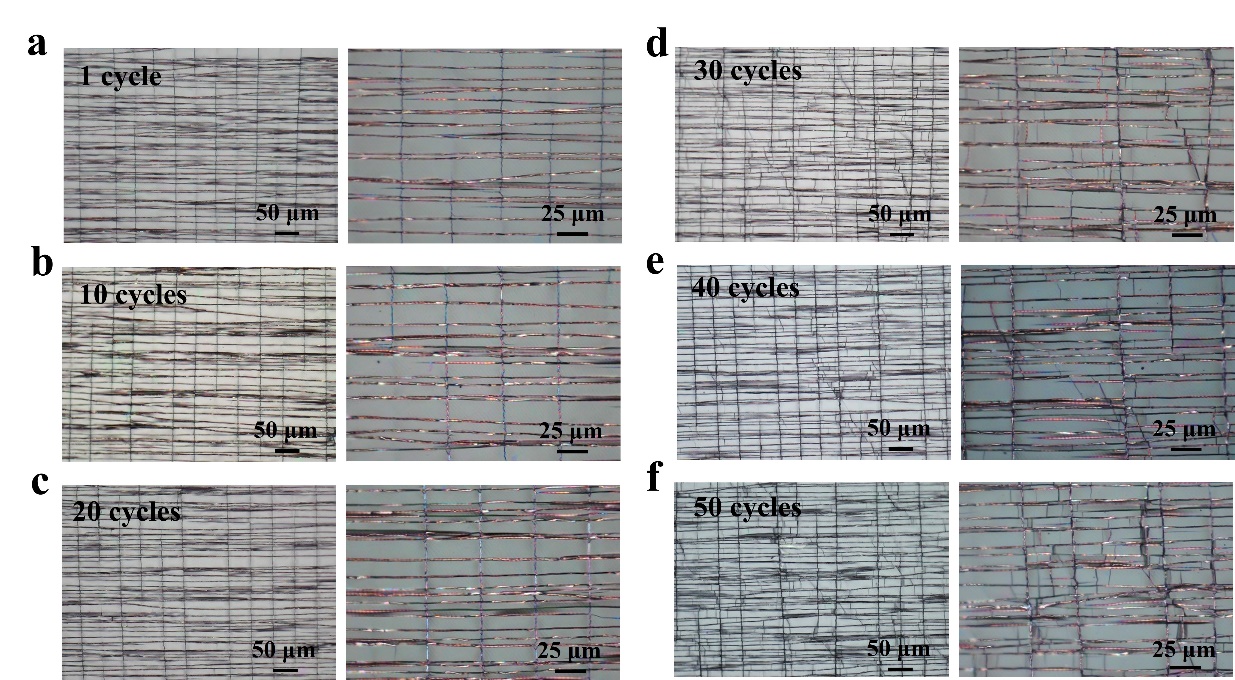


**Figure S32.** The evolution of surface morphology of the folding surface upon repeated stretching and releasing. (a-f) Optical microscopy images of the folding structure after different stretching and releasing cycles. (a) 1 cycle; (b) 10 cycles; (c) 20 cycles; (d) 30 cycles; (e) 40 cycles; (f) 50 cycles.


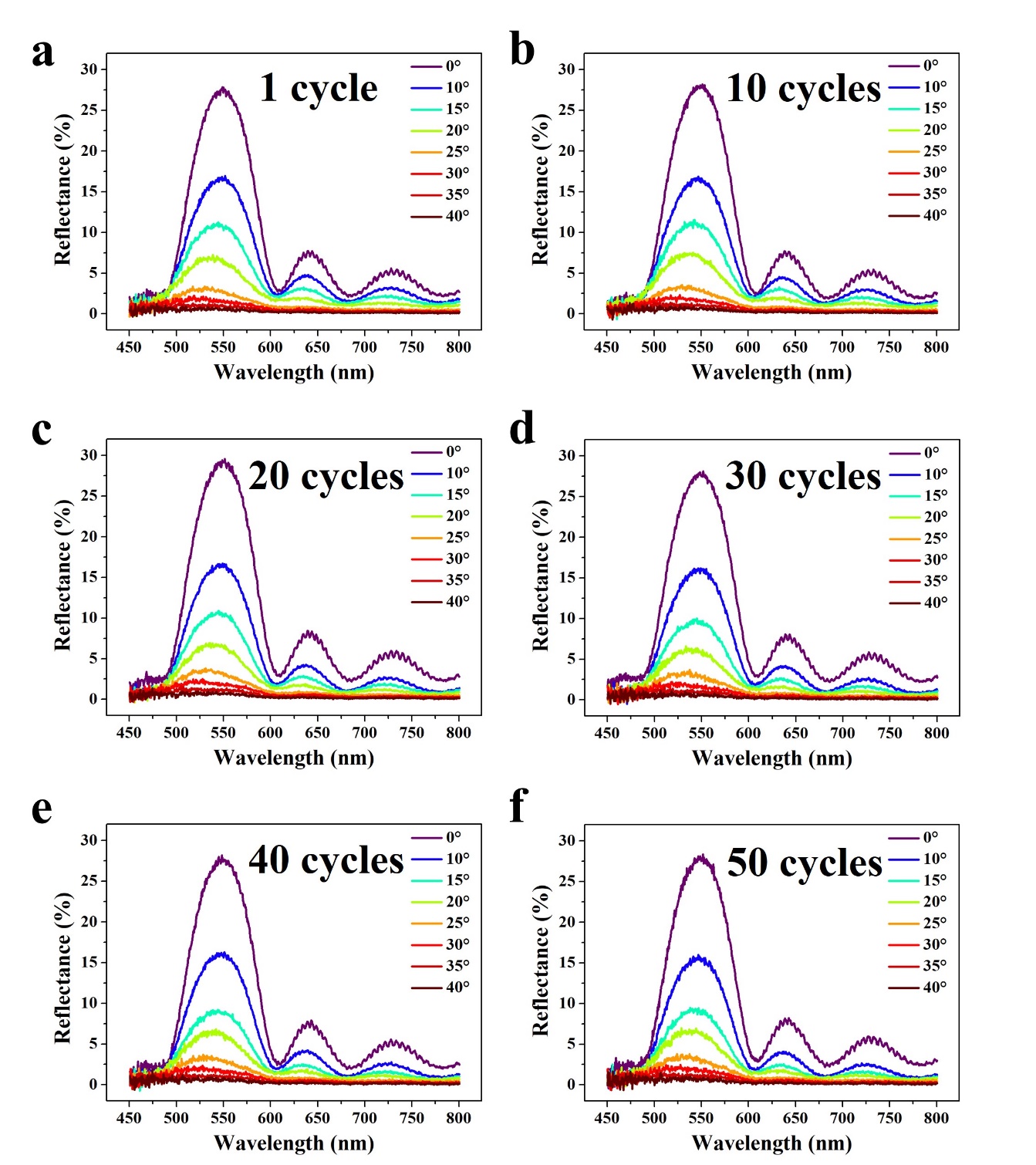


**Figure S33.** Optical properties of the photonic superstructure upon repeated stretching and releasing of the folding structure. (a-f) Reflectance spectra measured after different stretching and releasing cycles of the folding structure. (a) 1 cycle; (b) 10 cycles; (c) 20 cycles; (d) 30 cycles; (e) 40 cycles; (f) 50 cycles (*α* = 0°, *β* = 0-40°).


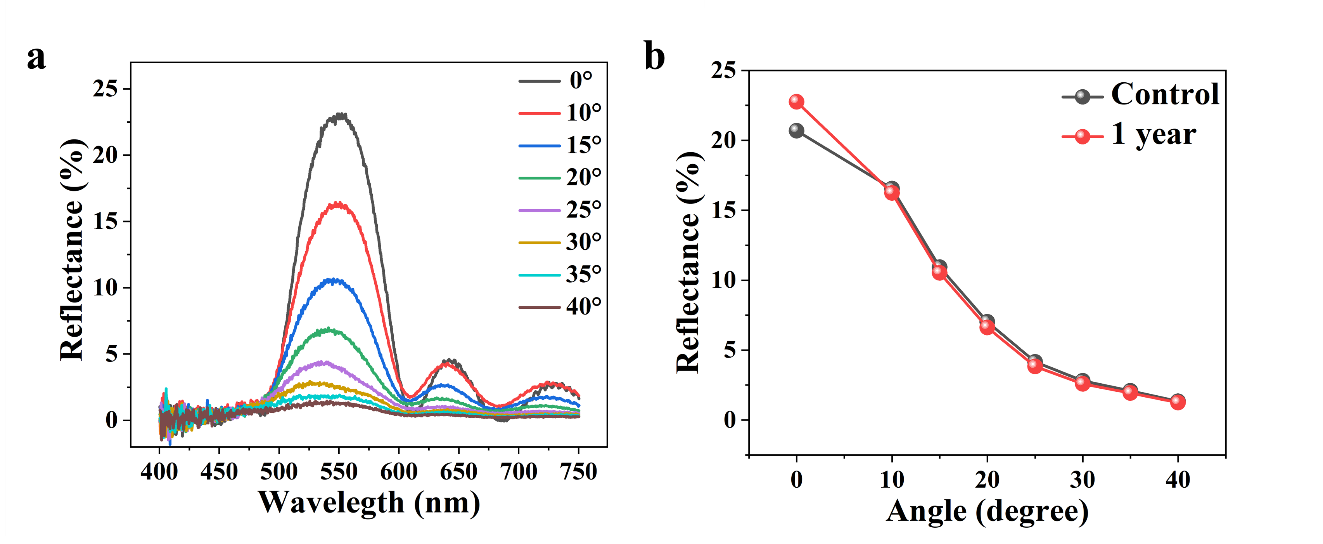


**Figure S34.** Long-lasting stability of the folding structure. (a) Reflectance spectra of the photonic superstructure assembled using the 1-year-old folding sample measured under diffusive reflection mode with various detection angles (β). (α = 0°, γ = 0°) (b) comparison of reflectance at different detection angles of the freshly prepared folding (control) and the 1-year-old sample.


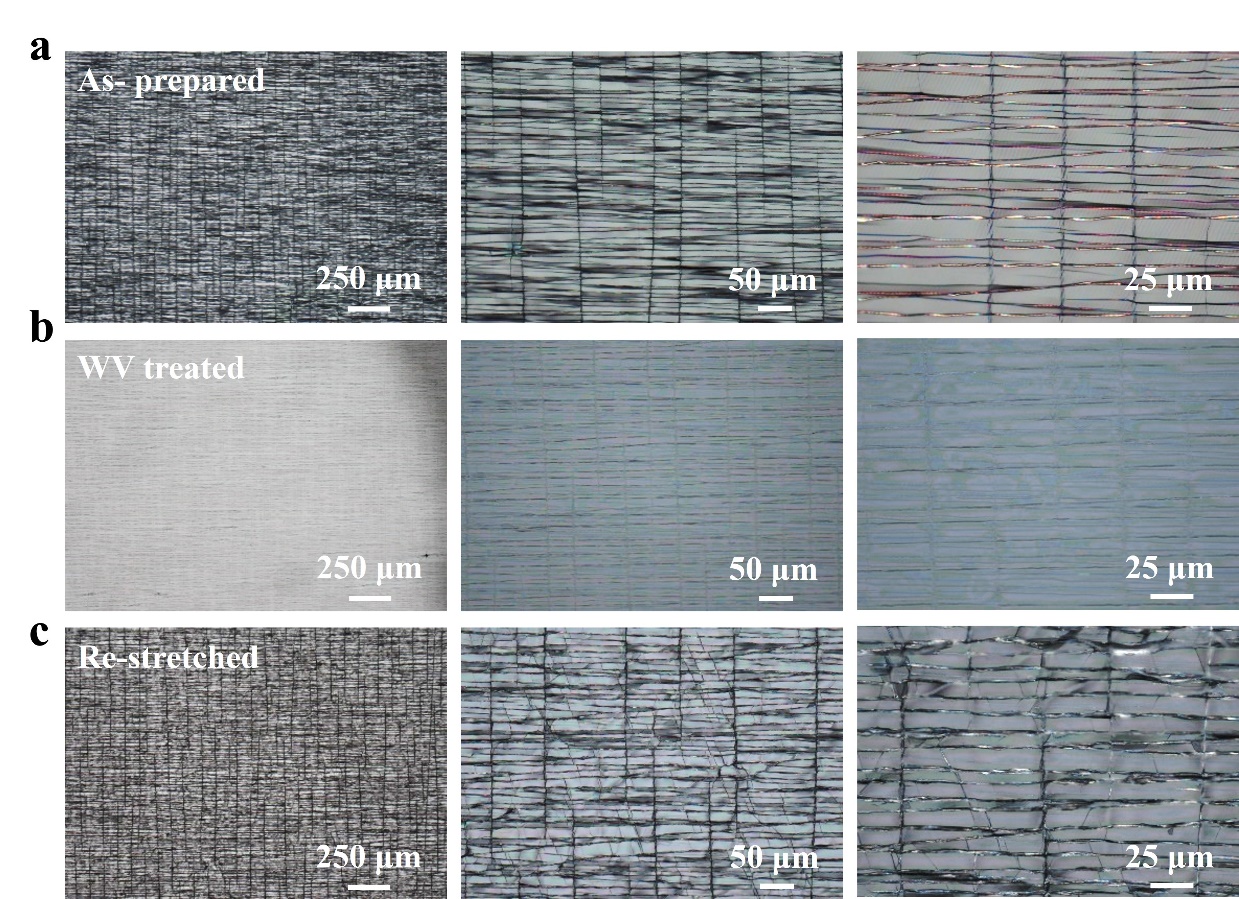


**Figure S35.** Folding structure erasure induced by water vapor (WV) exposure. a-c, Optical microscopy images showing the reversible folding/unfolding process. (a) As-prepared folding surface; (b) WV-treated bilayer; (c) Re-stretched folding surface. The folding patterns can be easily erased by water vapor exposure and reformed by reapplying mechanical strain.


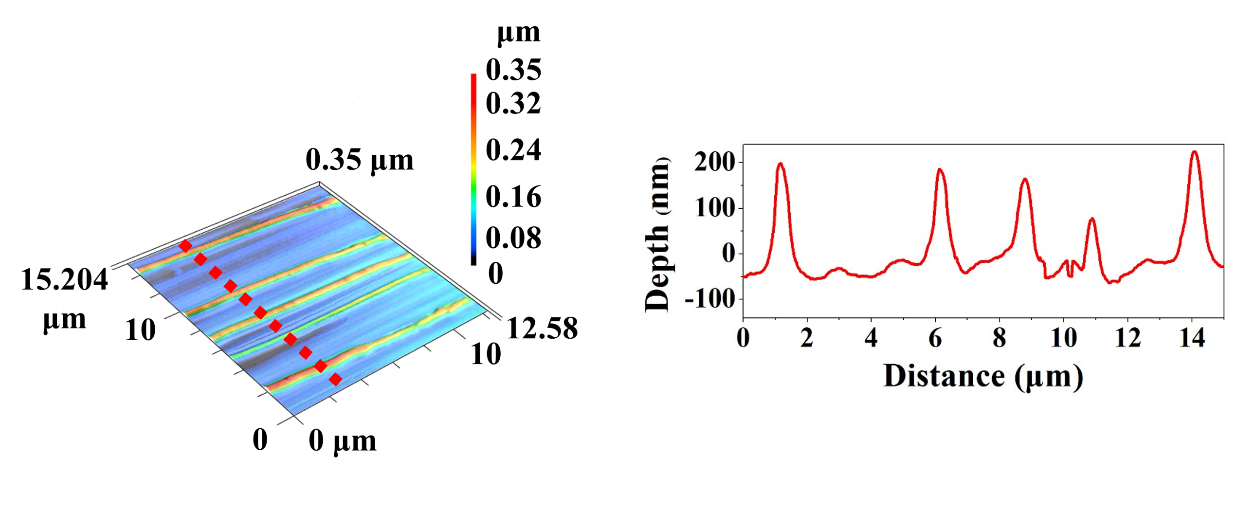


**Figure S36.** The effect of WV treatment on the folding geometry. 3D LSCM image of the WV-erased region of folding surface (left) and corresponding cross-section profile (right). The heights of the folds dramatically decrease after WV exposure.


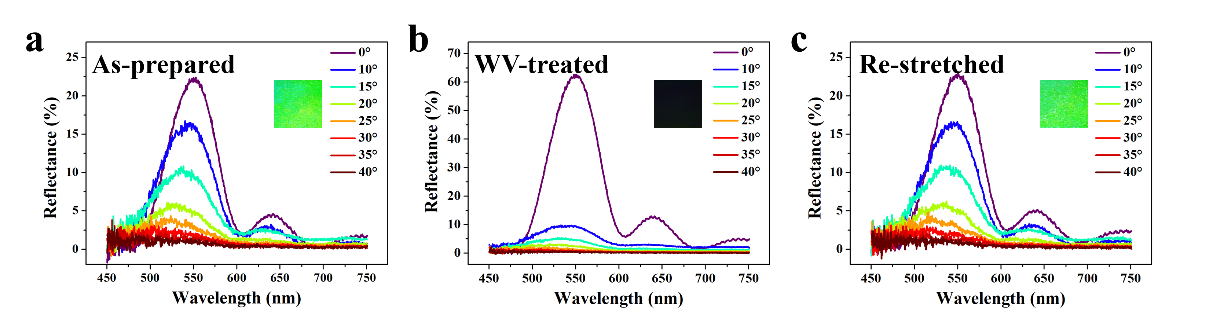


**Figure S37.** The influence of WV treatment on the optical properties of the photonic superstructure. (a-c) Reflectance spectra of as-prepared (a), water-vapor-treated (b), and re-stretched (c) samples at varying detecting angles (*β*) (*α* = 0°, *γ* = 0°). The inset in each panel shows the corresponding optical image captured at *β* = 0° (*α* = 20°, *γ* = 0°). Water vapor exposure results in decreased diffuse reflection and increased specular reflection.


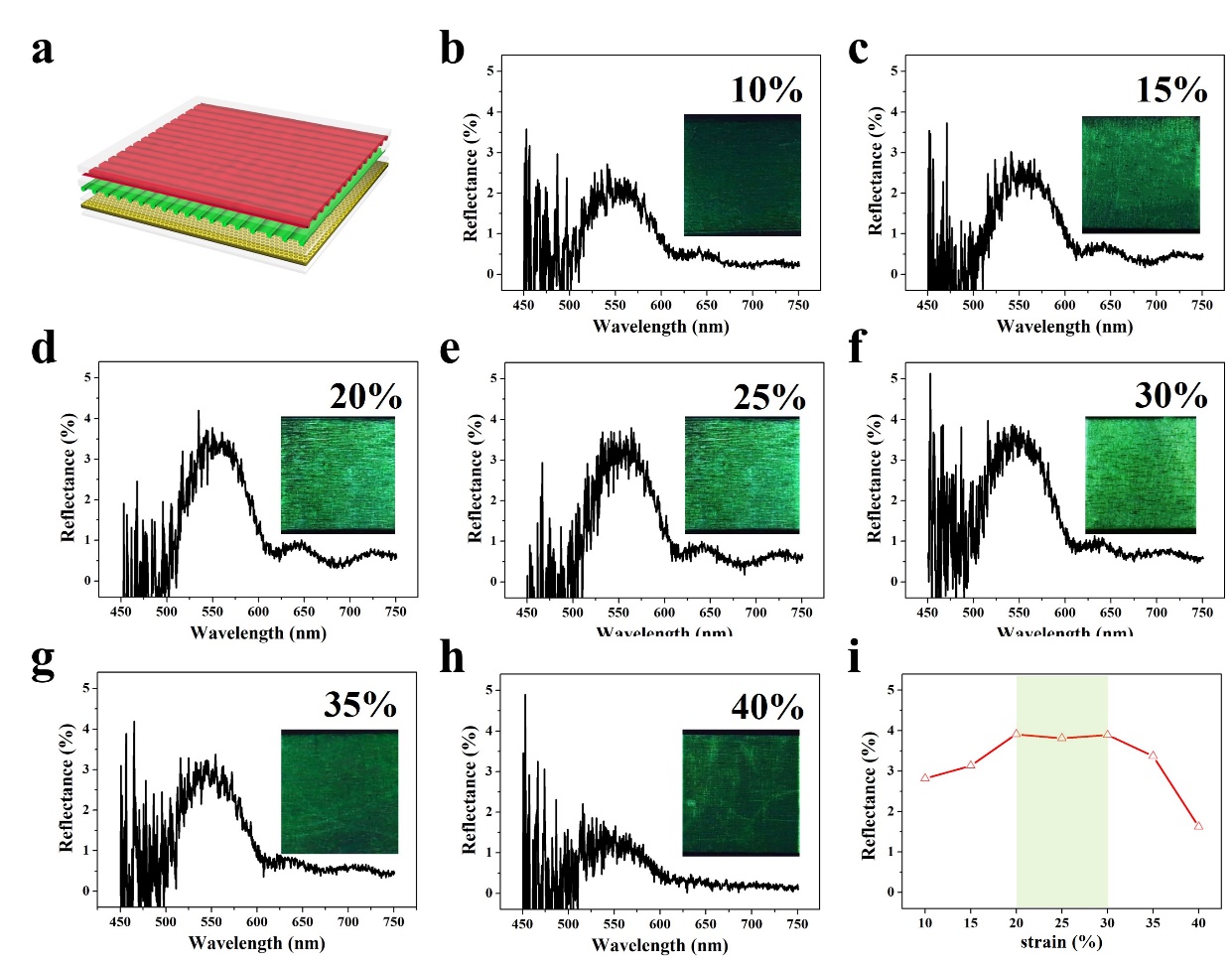


**Figure S38.** Optical properties of the photonic superstructure with two orthogonally superposed folding surfaces of identical topography. (a) Schematic of the photonic superstructure. (b-h) Reflectance spectra under different mechanical strains. Insets show the corresponding photographs. (i) Dependence of reflectance on the applied strain. The shadowed area displays a strain range with high reflectivity.


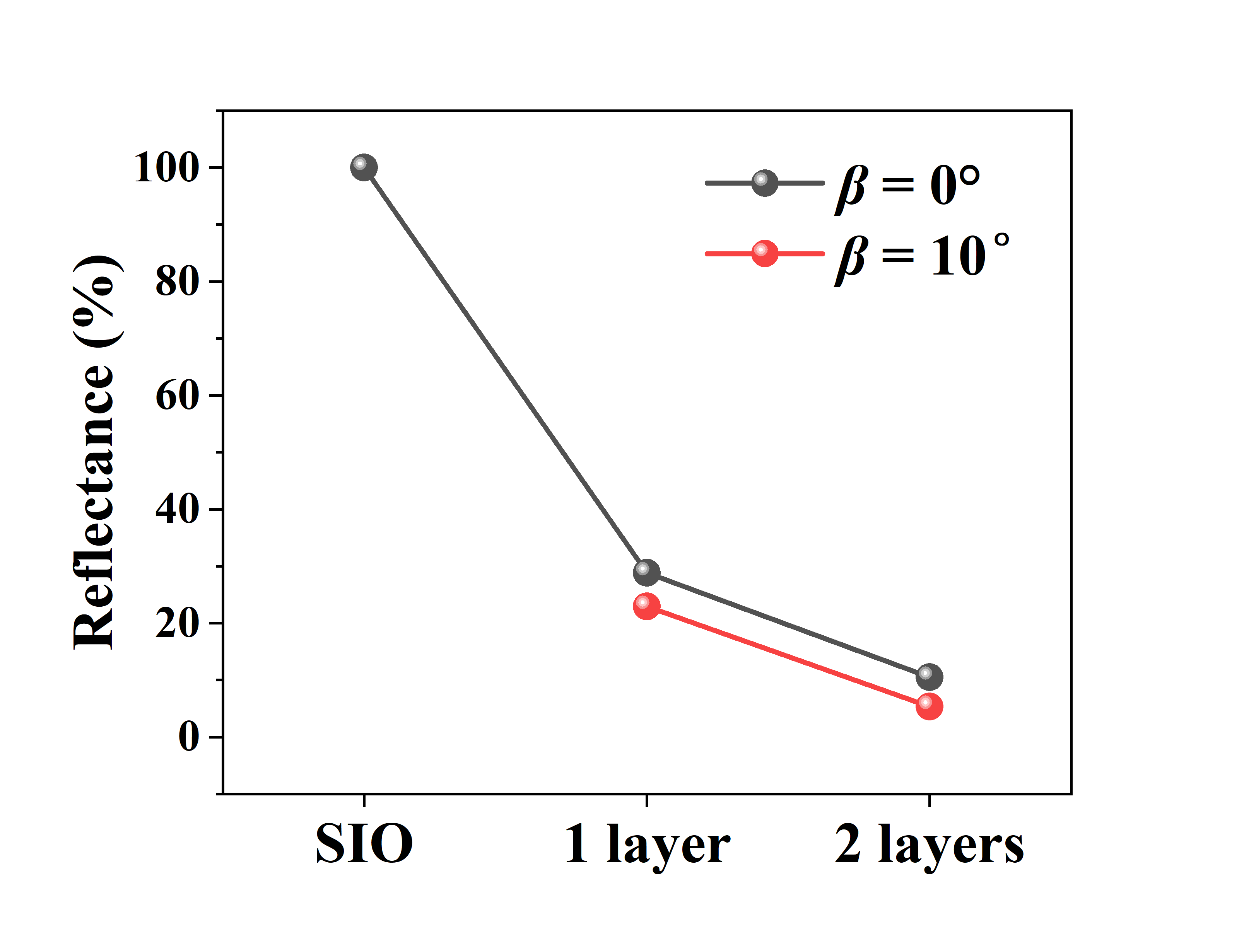


**Figure S39.** Comparison of the reflectance of SIO, SIO with one folding layer, and SIO with two orthogonally superposed folding layers. The reflectance of SIO is normalized for comparison.


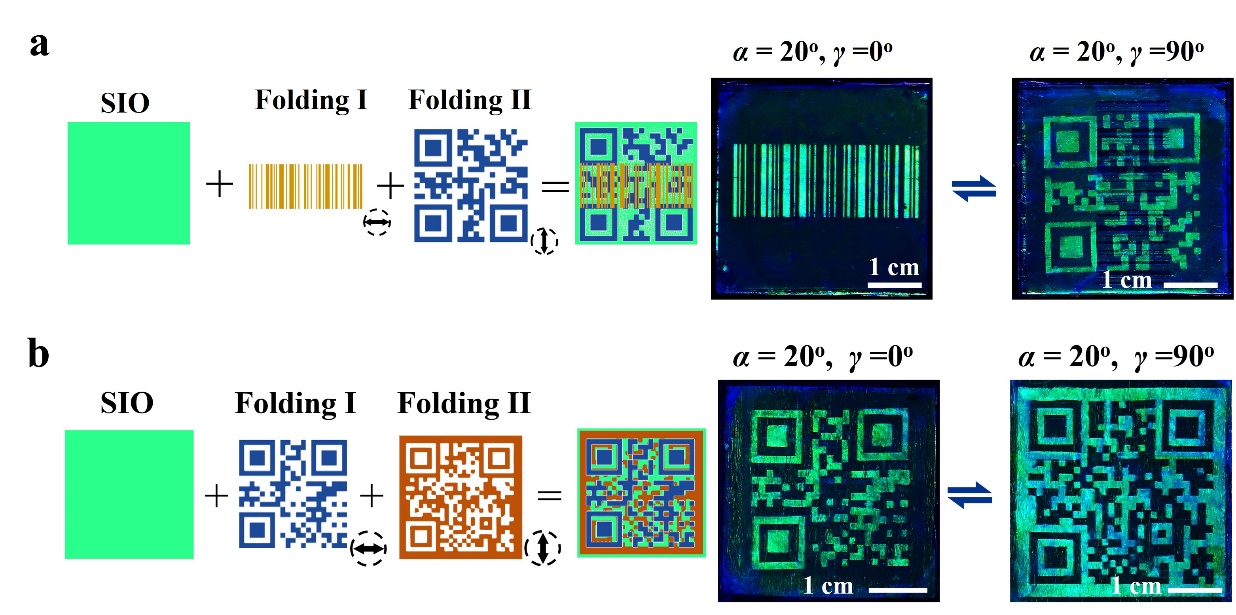


**Figure S40.** Image transformation between different codes. (Left) Schematic of photonic device design for transformation between 1D and 2D code patterns (a) or two 2D codes (b). (Right) Optical images showing the transition from a 1D to a 2D code pattern (a) or from a 2D to another 2D code pattern upon changing the horizontal rotation angle *γ* from 0° to 90° (*α* = 20°, *β* = 0°).

**Supplementary Movie Legends**

**Movie S1:** Photonic superstructure with a butterfly folding pattern captured under various illumination-viewing modes. The butterfly pattern becomes visible only under diffusive illumination when the incident light is perpendicular to the folding orientation.

**Movie S2:** Transformations in images with complex patterns. The change of horizontal orientation angle (*γ*) triggers the switching between the dragon pattern and the phoenix pattern. (*α* = 20°, *β* = 0°)

**Movie S3:** “modularized” multidirectional imaging. The modulation of light illumination direction induces the reversible switching of the oracle bone inscriptions of the twelve Chinese zodiacs, the animal patterns, and the corresponding twelve terrestrial branches.

**Movie S4:** Information transformation between two 2D codes or between 1D and 2D codes induced through the manipulation of horizontal orientation angle (*γ*). (*α* = 20°, *β* = 0°)

**Movie S5:** Extraction of numerical data by altering illumination direction. The deciphered data can be used to create a secret code, which can then be employed in the development of a high-level encryption system.

**Reference**

[1] F. Xia, L. Jiang, *Adv. Mater.* **2008**, *20*, 2842.
